# 5-Iodotubercidin sensitizes cells to RIPK1-dependent necroptosis by interfering with NFκB signaling

**DOI:** 10.1038/s41420-023-01576-x

**Published:** 2023-07-26

**Authors:** Chanchal Chauhan, Andreas Kraemer, Stefan Knapp, Mark Windheim, Alexey Kotlyarov, Manoj B. Menon, Matthias Gaestel

**Affiliations:** 1 Institute of Cell Biochemistry, Hannover Medical School, Hannover, 30625 Germany; 2 Institute of Pharmaceutical Chemistry, Goethe University Frankfurt am Main, 60438 Frankfurt am Main, Germany; 3 Structural Genomics Consortium (SGC), Buchmann Institute for Life Sciences (BMLS), Goethe University Frankfurt am Main, 60438 Frankfurt am Main, Germany; 4 Frankfurt Cancer Institute (FCI) and German Translational Cancer Network (DKTK) site Frankfurt–Mainz, 60438 Frankfurt am Main, Germany; 5 Kusuma School of Biological Sciences, Indian Institute of Technology Delhi, New Delhi, 110016 India

**Keywords:** Stress signalling, Phosphorylation

## Abstract

Receptor-interacting protein kinases (RIPK)-1 and -3 play crucial roles in cell fate decisions and are regulated by multiple checkpoint controls. Previous studies have identified IKK1/2- and p38/MK2-dependent checkpoints that phosphorylate RIPK1 at different residues to inhibit its activation. In this study, we investigated TNF-induced death in MAPK-activated protein kinase 2 (MK2)-deficient cells and found that MK2 deficiency or inactivation predominantly leads to necroptotic cell death, even without caspase inhibition. While RIPK1 inhibitors can rescue MK2-deficient cells from necroptosis, inhibiting RIPK3 seems to switch the process to apoptosis. To understand the underlying mechanism of this switch, we screened a library of 149 kinase inhibitors and identified the adenosine analog 5-Iodotubercidin (5-ITu) as the most potent compound that sensitizes MK2-deficient MEFs to TNF-induced cell death. 5-ITu also enhances LPS-induced necroptosis when combined with MK2 inhibition in RAW264.7 macrophages. Further mechanistic studies revealed that 5-ITu induces RIPK1-dependent necroptosis by suppressing IKK signaling in the absence of MK2 activity. These findings highlight the role for the multitarget kinase inhibitor 5-ITu in TNF-, LPS- and chemotherapeutics-induced necroptosis and its potential implications in RIPK1-targeted therapies.

## Introduction

The TNF receptor-interacting protein kinases (RIPK)-1 and -3 regulate the cell fate in response to diverse stimuli and are subjected to multiple checkpoint controls (reviewed in [1, 2]). RIPK1 is recruited to the TNF receptor or related death-receptors and modulates pro-survival gene expression, inflammation and cell death signaling. Receptor ligand-binding induces subsequent ubiquitination of RIPK1, promoting the formation of the signaling complex I, which predominantly modulates pro-survival signaling [3]. Receptor-associated RIPK1 in complex I is required for the activation of MAP kinases and NFκB-mediated pro-survival gene expression. Activated RIPK1 may subsequently be released from the receptor to form a cytosolic complex containing CASP8 and FADD to mediate apoptotic cell death (signaling complex IIb) [3, 4]). Only in the absence of CASP8, RIPK3 can be recruited to RIPK1 inducing subsequent MLKL phosphorylation, oligomerization and necroptosis [5–7]. RIPK1 is also involved in cell death and inflammatory signaling downstream to the interferon-alpha (IFNαR) [8] and Toll-like receptors (TLR)−3 and −4 [9, 10]. Treatment of myeloid cells with TLR4-activating bacterial lipopolysaccharide (LPS) in combination with the pan-caspase inhibitor zVAD-fmk (zVAD) results in RIPK1-dependent necroptosis [10]. In response to genotoxic stress and depletion of inhibitors of apoptosis proteins, RIPK1 also assembles into the ripoptosome, a high molecular weight cytotoxic protein complex, inducing cell death independent of receptor ligation [11].

The above findings put RIPK1 to the center of a signaling hub, which determines the outcome of cell fate decisions in the context of TNF, TLR and genotoxic signaling. A complex array of post-translational modifications (PTMs) of RIPK1 determines the balance between cell death and pro-survival signaling (reviewed in [2, 12]). Receptor-associated ubiquitination of RIPK1 is essential for recruiting MAP3K7/TAK1 and IKKα/β/γ leading to MAPK- and NFκB pathway activation, respectively. Deubiquitinated RIPK1 undergoes autophosphorylation, with S166 in its activation loop being the best-characterized autophosphorylation site associated with kinase activation. Various protein kinases have been implicated in phosphorylating RIPK1 and regulating the transition from pro-survival signaling to kinase activation and subsequent assembly of cytotoxic complexes in the cytoplasm, including the ripoptosome, complex IIb and the necrosome. Apart from their canonical role in the activation of pro-survival NFκB signaling, the inhibitor of κB (IκB)-kinases IKKα/IKK1 and IKKβ/IKK2 have been demonstrated to phosphorylate RIPK1 in complex I, thereby suppressing RIPK1 activation and assembly of complex IIb [13]. In addition, the cytoplasmic phosphorylation of RIPK1 at serines S321 and S336, mediated by MAPK-activated protein kinase 2 (MK2), acts as a second checkpoint that inhibits both, RIPK1-S166 autophosphorylation and the assembly of complex IIb [14–16]. In addition to IKK1/2-mediated phosphorylation at S25 [17] and MK2-mediated phosphorylation at S320 and S335 [13–15], recent studies have demonstrated that TBK1/IKKε phosphorylates RIPK1 at T189 [18] and JAK1/SRC phosphorylates RIPK1 at Y384 [19], resulting in suppression of the cytotoxic response. Furthermore, there is emerging evidence indicating that PPP1R3G/PP1γ-mediated dephosphorylation counteracts kinase-mediated suppression of RIPK1-dependent apoptosis and necroptosis [20]. Although the mechanisms underlying the modulation of intra- and intermolecular interactions and S166 autophosphorylation by these PTMs are still unknown, it has become evident that interfering with these RIPK1-kinases using small-molecule inhibitors sensitizes cells to TNF-induced cell death. While the sole loss of MK2 does not strongly enhance TNF-induced cytotoxicity, its combination with IKK inhibitors has revealed an MK2-dependent checkpoint in LPS-zVAD-induced necroptosis [14]. In addition, inhibition of p38/MK2 facilitates ripoptosome assembly and autocrine TNF production in myeloid cells induced by smac mimetics (SM), and has been suggested as a strategy to overcome SM-resistance in leukemia [16, 21, 22].

5-Iodotubercidin (5-ITu) is a halogenated pyrrolopyrimidine and nucleoside analog and has been reported as a potent inhibitor of adenosine uptake and adenosine kinase activity [23]. While originally used as an adenosine kinase inhibitor, later studies revealed its potential as a pan-protein kinase inhibitor [24]. 5-ITu was shown to enhance human β-cell proliferation and glucose-dependent insulin secretion by targeting DYRK1A kinase [25]. Low concentrations of 5-ITu also inhibit Haspin/GSG2, a histone-H3 kinase [26–28]. It has also been described as a potent chemotherapeutic agent with strong antitumor activity [29]. Recently, 5-ITu has been demonstrated to inhibit SARS-CoV2 replication via an adenosine kinase-dependent mechanism [30].

The switch between pro-survival and cytotoxic functions of RIPK1 is regulated by diverse PTMs, acting as checkpoints for RIPK1 activation [31]. To understand the MK2-dependent checkpoint on RIPK1 autophosphorylation and the assembly mechanisms of complex IIb and necrosome, we analyzed the mode of cell death in MK2-deficient cells. Interestingly, MK2 deficiency is not only associated with enhanced RIPK1 activation, but also exerts a predominantly necroptotic mode of cell death. In a screen for small-molecule inhibitors specifically sensitizing MK2-deficient cells to TNF-induced necroptosis, we identified 5-ITu as a prominent regulator of RIPK1-dependent cell death. We present evidence that 5-ITu acts as a modulator of the NFκB pathway and, therefore, compromises the IKK-dependent checkpoint of RIPK1 activation. Our findings indicate that 5-ITu and MK2 inhibition could have additive effects in sensitizing cells to RIPK1-dependent necroptosis.

## Results

### MK2-KO and MK2/3-DKO MEFs predominantly undergo necroptosis rather than apoptosis

**Fig. 1:**
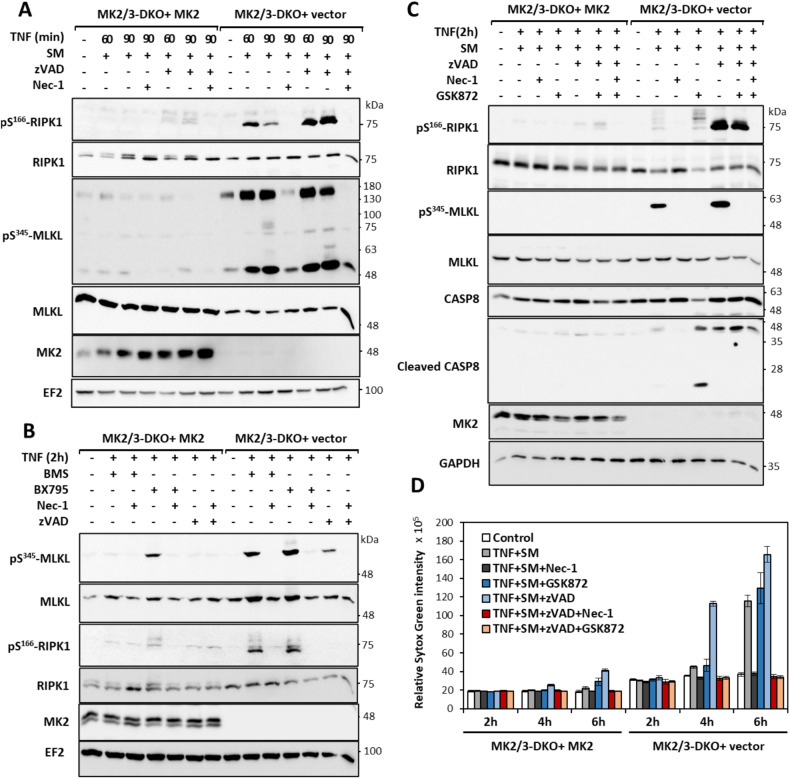
MK2-deficient cells predominantly undergo necroptosis in response to pro-apoptotic stimuli. **A** MK2/3-deficient MEFs transduced with MK2 expression or control vector are treated with TNF in the presence of smac mimetics (SM), a pro-apoptotic stimulus, for 60 and 90 min. **B** Diverse apoptotic stimuli including combinations of TNF with the IKK1/2 inhibitor BMS345541 (BMS) and with BX795 (TBK1 inhibitor) induce predominant necroptotic response in MK2/3-deficient cells. **C** MK2/3-deficient cells were pre-treated with RIPK3 inhibitor (GSK872) and SM for 30 min followed by TNF treatment for 2 h. **A**–**C** Numbers at the right of the blot indicate molecular mass of the marker proteins in kDa. **D** Sytox Green-based cytotoxicity assay was performed with MK2/3-deficient and MK2-rescued cells treated in the presence and absence of caspase inhibitor (zVAD) as indicated.

Previous studies have identified MK2-mediated RIPK1 phosphorylation as a checkpoint of TNF- and Toll-like receptor-mediated RIPK1 activation [14–16]. TNFα stimulation in the presence of SM or IKK1/2 inhibitors were shown to sensitize MK2-deficient cells to RIPK1 activation-dependent cytotoxicity. Inactivation of the IKK1/2-dependent checkpoint seems to be upstream to the MK2-dependent checkpoint [14–16]. To further understand the mechanism of cell death in MK2/3-deficient cells, we monitored RIPK1 activation and signaling in these cells. Consistent with previous studies, MK2/3-deficient mouse embryonic fibroblasts (MEFs) showed higher levels of RIPK1 autophosphorylation (pRIPK1-S166) upon stimulation with TNF and SM, compared to the same cells but rescued with retrovirally expressed MK2 (Fig. 1A). As expected, pS166-RIPK1 was further enhanced when the cells were co-treated with the pan-caspase inhibitor zVAD-fmk to induce the switch to necroptosis. Surprisingly, there were significantly higher levels of the necroptotic marker represented by phosphorylated and oligomerized MLKL (pS345-MLKL) detected in the DKO cells, even in the absence of caspase inhibition. This indicates that MK2/3-deficiency is associated with predominant necroptosis in response to a pro-apoptotic stimulus. Similar results were observed when MK2-deficient MEFs were used instead of MK2/3-DKO cells (Supplementary Fig. S1A). In addition to MK2 being a RIPK1 kinase in the cytoplasm, we have also demonstrated a strong association between MK2 and RIPK1 in ripoptosome-like complexes in transfected cells [14]. To rule out any differences between the absence of MK2 protein and the absence of MK2 activity in the observed phenotype, we performed similar experiments in wild-type (WT) MEFs in the presence and absence of the MK2 inhibitor PF3644022. Consistent with the data from the knockout cells, MK2 inhibition also induced a predominant necroptotic response (Supplementary Fig. S1B).

We then asked the question whether this pro-necroptotic response in the absence of the MK2 checkpoint is specific to SM treatment. When the IKK1/2 inhibitor BMS345541 (BMS) or the TBK1 inhibitor BX795 were combined with TNFα to provide different pro-apoptotic stimuli, we observed a stronger necroptotic response in the absence of MK2/3 again, as indicated by RIPK1 and MLKL phosphorylation (Fig. 1B). In all conditions analyzed, RIPK1 and MLKL phosphorylation were dependent on RIPK1 activity and inhibited by RIPK1 inhibitor necrostatin-1 (Nec-1) (Fig. 1A, B and Supplementary Fig. S1A, B). It is known that MLKL-S345 phosphorylation is mediated by RIPK3 [7]. To understand the hyperactivation of the RIPK1-RIPK3-MLKL axis in response to the pro-apoptotic TNF-SM stimulus, we pre-treated the cells with the RIPK3 inhibitor GSK872. Interestingly, inhibition of RIPK3 led to a significant increase of cleaved caspase-8 (CASP8) in TNF-SM-treated MK2/3-deficient cells, indicating a switch to apoptosis (Fig. 1C). The observations made by immunoblot analyses of necroptotic and apoptotic markers were verified by Sytox Green-based cytotoxicity assays, wherein MK2/3-deficiency was associated with strong necrotic death in the presence and absence of the caspase inhibitor (Fig. 1D). While Nec-1 protected cells independent of the type of cytotoxic stimulus, the RIPK3 inhibitor (GSK872) was effective only in combination with caspase inhibitor (zVAD).

### A screen for small molecules sensitizing MK2-deficient cells to cell death

**Fig. 2:**
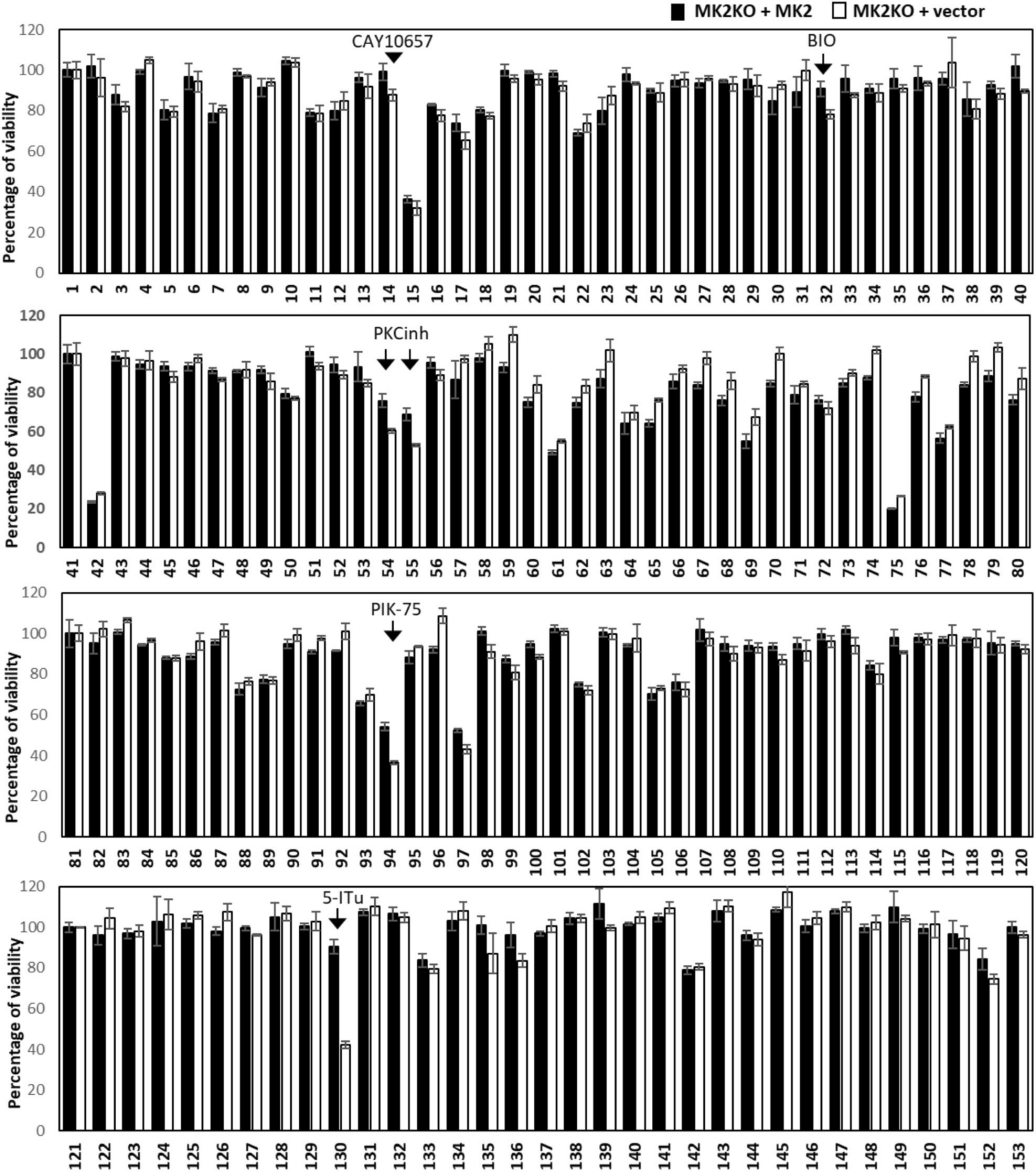
A screen for small molecules facilitating TNF-induced death in MK2-deficient cells. MK2-deficient MEFs transduced with MK2 expression or control vector were treated with TNF alone or in combination with inhibitors from the kinase inhibitor panel (at 10 µM concentration) for 6 h. Cell viability was quantified by the CCK8 colorimetric assay. Each treatment was performed in triplicates. While TNF alone is not cytotoxic, several small molecules sensitize both MEF lines to TNF (comps. 15, 42, 133, 142). Compound 14/CAY10657 (IKK2 inhibitor), comp. 32/BIO (GSK3 inhibitor), comp. 54,55/Bisindolylmaleimide VIII/IX (PKC inhibitors), comp. 94/PIK-75 (PI3 kinase inhibitor) and comp. 130/5-Iodotubercidin (5-ITu) display specific sensitizing effects dependent on MK2 deficiency. Average values of *n* = 3 independent wells are plotted ± SD.

The observed sensitivity of MK2-deficient cells to RIPK1-dependent cell death is attributed to the lack of MK2-mediated phosphorylation of RIPK1 in the intermediate domain, which suppresses RIPK1 activation and autophosphorylation in the activation loop at residue S166. However, this does not explain the predominant necroptotic death associated with MK2 deficiency. To understand the pathways involved in this process, we aimed to identify additional small molecules that sensitize MK2-deficient cells to TNF-dependent/RIPK1-dependent cell death. Therefore, we conducted a screen using a library of characterized small-molecule kinase inhibitors (Supplementary Table S1). In this screen, MK2-KO cells transduced with an empty vector or rescued with an MK2 expression vector were treated with TNFα alone or together with the inhibitors of the screening panel for 6 h, and cell viability was quantified by Cell Counting Kit-8 (CCK8) colorimetric assay (Fig. 2). Consistent with previous findings, TNF alone was not cytotoxic; however, several small molecules sensitized both MEF lines to TNF treatment (compound 15, 42, 133, 142). More interestingly, CAY10657 (IKK2 inhibitor, comp.14), BIO (GSK3 inhibitor, comp. 32), Bisindolylmaleimide VIII (comp. 54) and IX (comp. 55, PKC inhibitors), PIK-75 (PI3 kinase inhibitor, comp. 94) and 5-Iodotubercidin (5-ITu, comp.130) displayed a specific sensitizing effect in the absence of MK2 (Fig. 2 and Supplementary Table S2). The strongest and most significant effect was observed with 5-ITu, an adenosine derivative that acts both as adenosine kinase inhibitor and as protein kinase inhibitor [24, 32, 33].

### 5-ITu sensitizes MK2-KO cells to RIPK1-dependent cell death similar to SM and IKK inhibition

**Fig. 3:**
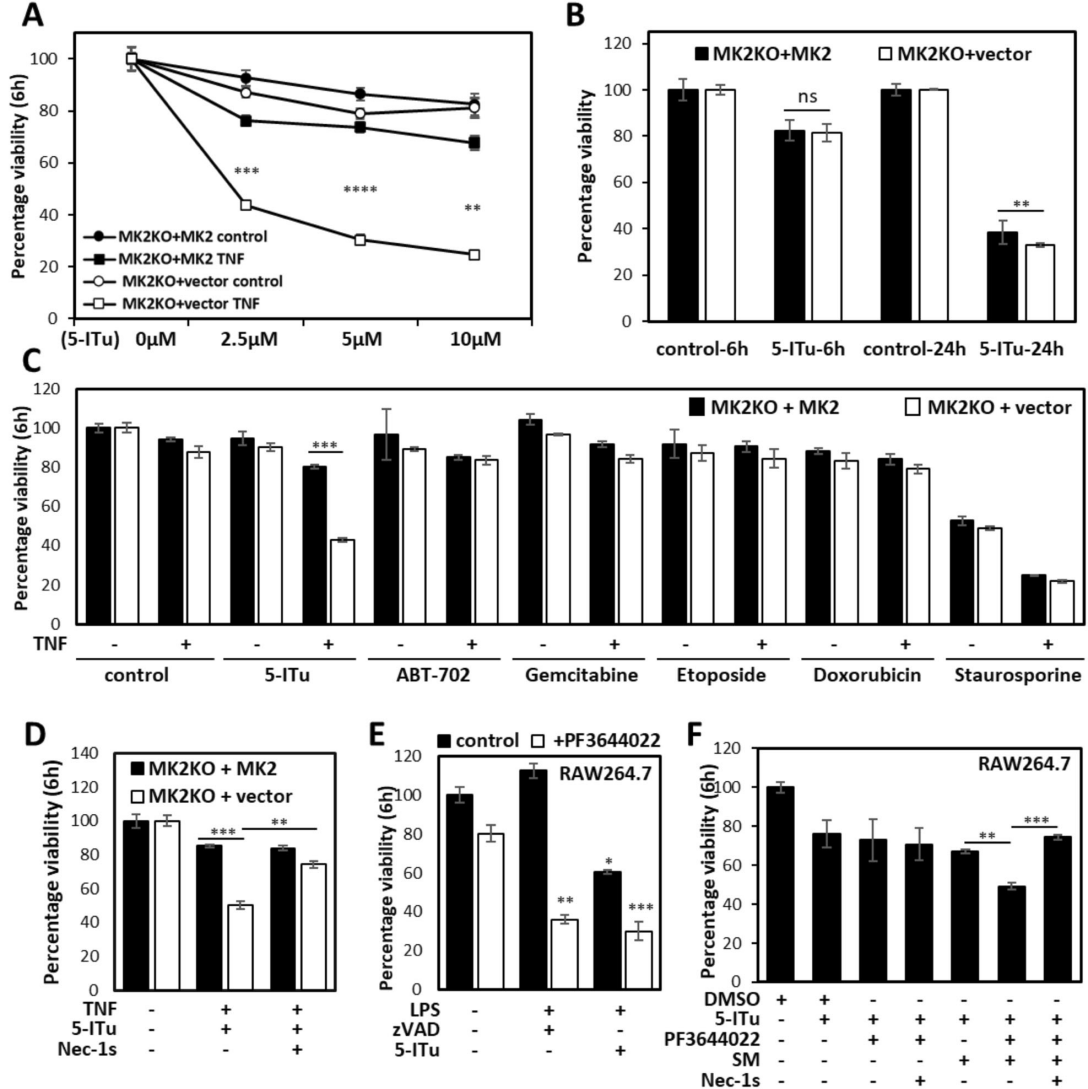
5-ITu potentiates RIPK1-dependent cell death in the absence of MK2 activity. **A** MK2-deficient MEFs transduced with MK2 expression or control vector were treated with different doses of 5-ITu in the presence or absence of TNF (10 ng/ml) for 6 h. **B** Cells of the indicated genotype were treated with 10 µM 5-ITu for 6 and 24 h and cell viability was quantified. **C** MK2-deficient MEFs transduced with MK2 expression or control vector were treated with indicated small molecules (5 µM ABT-702, 100 nM gemcitabine, 5 µM etoposid, 5 µM doxorubicin and 5 µM staurosporine) in the presence or absence of TNF for 6 h and cell viability was assessed. **D** MK2-deficient MEFs transduced with MK2 expression or control vector were treated as indicated and viability was quantified after 6 h treatment. **E**, **F** RAW264.7 cells were treated as indicated to monitor the effect of 5-ITu in LPS-induced necroptosis (**E**) and SM-mediated autocrine TNF-dependent death (**F**). Average values of *n* = 3 independent wells are plotted ± SD (**p ≤ 0.001, ***p ≤ 0.0001, ****p ≤ 0.0001).

An effect of the adenosine analog 5-ITu on TNF signaling has not been reported so far. To further characterize and confirm the effect of 5-ITu on MK2/RIPK1-mediated cytotoxicity, we analyzed the impact of increasing doses of 5-ITu on sensitizing MK2 KO cells to TNF-induced cytotoxicity. When MK2-KO cells transduced with empty vector control or rescued with MK2 expressing vector were treated with 5-ITu and TNF, a dose-dependent loss of viability was observed only in the absence of MK2 (Fig. 3A). Similar results were observed when this experiment was performed with MK2/3-DKO MEFs rescued by MK2 or empty vector (Supplementary Fig. S2A). 5-ITU is a nucleoside analog with known cytotoxic effects. To understand whether the MK2-specific effects observed here represent general effects of 5-ITu-induced cell death, we monitored the cytotoxic effect of 5-ITu in the absence of TNF. While 5-ITu alone induced a general loss of cell viability of MEFs ranging from about 20% after 6 h of treatment to 60% after 24 h of treatment, no MK2 deficiency-associated phenotype could be observed (Fig. 3B). Even in the MK2/3-DKO background, the presence or absence of MK2 expression had no impact on cell death induced by 5-ITu alone (Supplementary Fig. S2B).

Since 5-ITu is a nucleoside analog and activator of DNA damage signaling, we decided to compare the effect of another nucleoside analog, gemcitabine, and of other DNA damage-inducing agents, etoposide and doxorubicin, on TNF-induced cytotoxicity. Unlike 5-ITu, none of these compounds induced significant cell death alone or in combination with TNF (Fig. 3C). We also tested the effect of the pan kinase inhibitor and apoptosis inducer staurosporine on TNF-induced cell death. While staurosporine induced significant loss of cell viability in the presence and absence of TNF, the effects were independent of MK2 expression (Fig. 3C). To determine whether the specific effect of 5-ITu on TNF-induced cell death is mediated by adenosine kinase inhibition, we utilized ABT-702 [34], a non-nucleoside adenosine kinase inhibitor. Neither the MK2-KO control, nor the MK2-rescued MEFs displayed significant loss of cell viability upon ABT-702 treatment alone or in combination with TNF (Fig. 3C). These results indicate that the MK2-specific effects exerted by 5-ITu on TNF-induced cell death is unique to this compound and cannot be explained by its general effects on DNA damage signaling, adenosine kinase inhibition or pan-protein kinase inactivation. Similar results were observed when we performed the experiments in the MK2/3-DKO MEFs (Supplementary Fig. 2C). Moreover, to monitor the RIPK1-dependence of the TNF-5-ITu-mediated cell death, we treated MK2-KO (Fig. 3D) and MK2/3-DKO (Supplementary Fig. 2D) MEFs and corresponding cells rescued with MK2 with TNF-5-ITu together with the RIPK1 kinase inhibitor necrostatin-1s (Nec-1s). Nec-1s markedly inhibited TNF-ITu-induced death in MK2 and MK2/3-deficient cells, clearly indicating the dependence of the observed cell death on RIPK1 activity.

### 5-ITu induces RIPK1-dependent macrophage cell death in combination with LPS and SM

In addition to the TNF receptor, TLR4 is also known to induce RIPK1-dependent cell death response, which is enhanced in the absence of MK2 activity [14]. To understand whether the effect of 5-ITu is specific to TNF signaling and MEFs, we monitored the effect of 5-ITu in LPS-induced necroptosis of RAW264.7 macrophages. When RAW264.7 cells were treated with the previously characterized necroptotic stimulus LPS-zVAD for 6 h, significant death was observed only in the presence of the MK2 inhibitor PF3644022 (Fig. 3E). Combined LPS-5-ITu-stimulation also led to significant loss of cell viability even in the absence of PF3644022, but the cytotoxicity was significantly enhanced by MK2 inhibition using PF364402 (Fig. 3E). We then asked the question whether 5-ITu may have any additive/synergistic effect with the SM-mediated autocrine TNF-dependent death in macrophages. Interestingly, we observed a similar MK2 inhibitor-dependent sensitizing effect of 5-ITu on the cell death induced by SM, which was rescued by the RIPK1 inhibitor Nec-1s (Fig. 3F). Again, 5-ITu alone displayed some reduction in the viability of cells after 6 h, which was neither MK2-, nor RIPK1-dependent.

### 5-ITu sensitizes cells to RIPK1-dependent necroptosis by downregulating IKK signaling

**Fig. 4:**
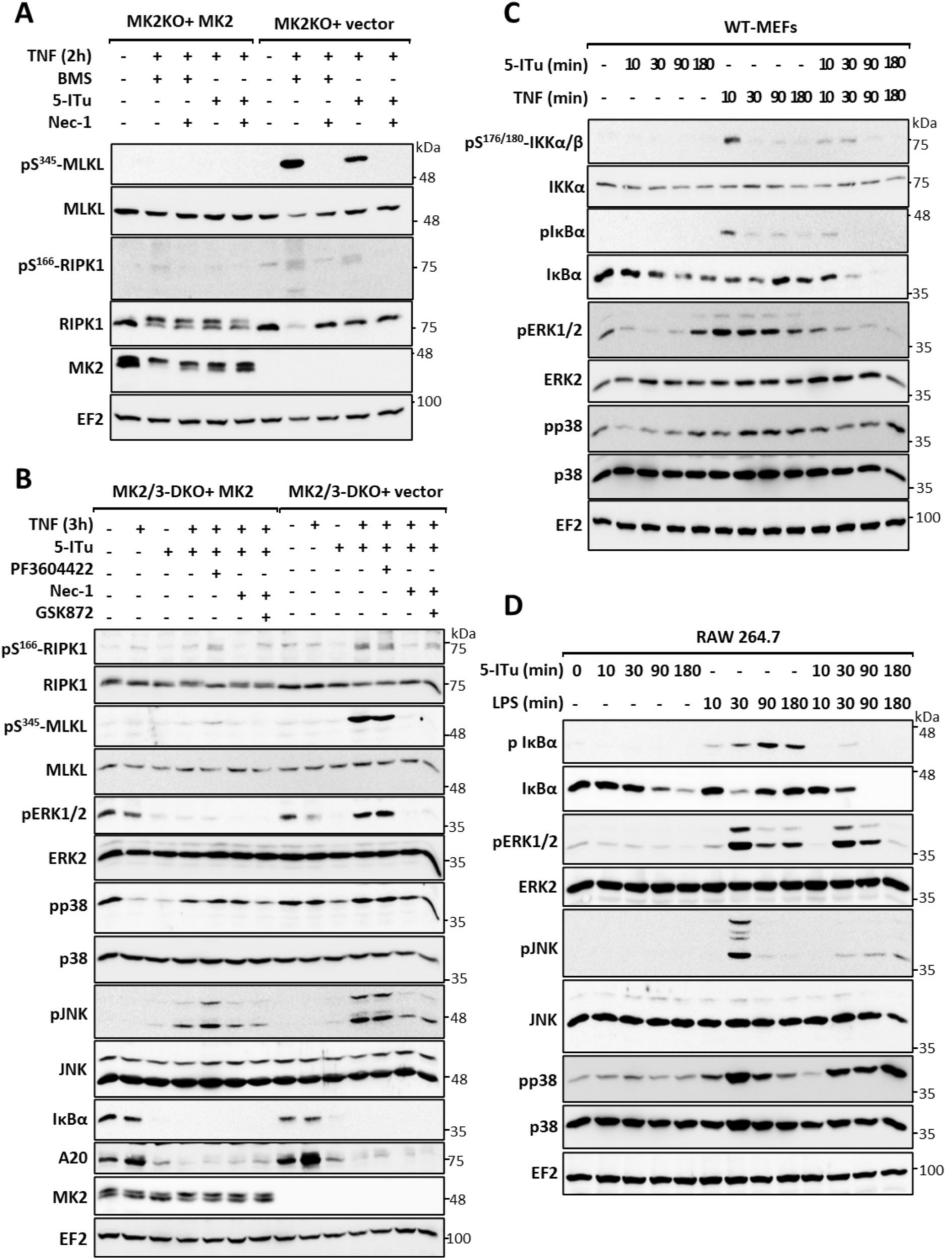
5-ITu sensitizes cells to RIPK1-dependent necroptosis by downregulating IKK signaling. **A** MK2-deficient MEFs transduced with MK2 expression or control vector were treated with IKK1/2 inhibitor BMS and 5-ITu in combination with TNF for 2 h. MLKL and RIPK1 phosphorylation were monitored with additional control blots. The RIPK1 band-shift induced by MK2-mediated phosphorylation is absent in MK2-KO cells. EF2 is shown as loading control. **B** MK2/3-deficient MEFs transduced with MK2 expression or control vector were treated with 5-ITu alone or in combination with TNF for 3 h. Cells were pre-treated for 30 min with the MK2 inhibitor PF3604422, the RIPK1 inhibitor Nec-1 or the RIPK3 inhibitor GSK872, as indicated. **C** Wild-type (WT) MEFs were treated with 5-ITu or TNF alone or in combination for short-term kinetics (10–180 min). **D** RAW264.7 cells were treated with 5-ITu or LPS alone or in combination for 10–180 min and signaling was monitored by immunoblotting as indicated. **A**–**D** Numbers at the right of the blot indicate molecular mass of the marker proteins in kDa.

To understand the mechanism by which 5-ITu sensitizes MK2-deficient cells to RIPK1-dependent cell death, we compared TNF-induced cell death signaling in the presence of IKK1/2 inhibitor BMS with the signaling in the presence of 5-ITu. In MK2-deficient cells, both BMS345541 and 5-ITu induced RIPK1 activation and the downstream event of necroptotic MLKL phosphorylation (Fig. 4A). This indicates, similar to SM and BMS345541, that 5-ITu-mediated cytotoxicity in the absence of MK2 activity is predominantly necroptotic. Then we conducted a detailed analysis of signaling in the MK2/3-DKO MEFs transduced with empty or MK2 expression vector. Consistent with previous experiments, there was prominent RIPK1 activation (pS166-RIPK1) and MLKL phosphorylation (pS345-MLKL) in the absence of MK2 activity (Fig. 4B). At the 3h-stimulation time-point analyzed, there was no detectable upregulation of the three canonical MAPK pathways with TNF alone and pERK1/2, pJNK and pp38 signals were mostly downregulated in stimulated cells. A20/TNFAIP3, a ubiquitin editing enzyme and NFκB feedback regulator was upregulated by TNF, downregulated by 5-ITu treatment and stayed low in response to all the cytotoxicity-inducing combinatory stimuli (Fig. 4B). The late-stage MAPK activation was predominantly downstream to necroptotic signaling as the activation corresponded to pMLKL and was inhibited by RIPK1 and RIPK3 inhibitors. Interestingly, 5-ITu treatment resulted in a complete depletion of IκBα protein, independent of TNF stimulation and RIPK1 activation, suggesting that this is a direct effect of 5-ITu (Fig. 4B).

To analyze the influence of 5-ITu on NFκB signaling in more detail, we performed short-term kinetics of 5-ITu and TNF alone and in combination in WT MEFs. As expected, TNF stimulation led to a transient increase in IKK1/2 activation and IκBα phosphorylation, which was blocked by 5-ITu (Fig. 4C). While 5-ITu led to a time-dependent decrease in IκBα levels, TNF treatment led to a transient decrease at 10–30 min, followed by increase probably owing to its re-synthesis. When cells were treated with a combination of 5-ITu and TNF, there was a complete loss of IκBα re-synthesis, which is a mark of NFκB pathway inhibition (Fig. 4C). These data clearly indicate that the 5-ITu-mediated effects on TNF signaling were caused by suppression of NFκB activity. To test whether this is also true for the LPS-mediated effects, we performed additional experiments in RAW264.7 macrophages. Thirty minutes of LPS stimulation resulted in the activation of all three MAPK, with JNK phosphorylation being more transient than pERK1/2 and pp38. While co-treatment with 5-ITu led to the suppression of JNK and ERK1/2 phosphorylation, a sustained p38 phosphorylation was detected when compared to LPS alone (Fig. 4D). In a similar manner as observed in MEFs, 5-ITu resulted in a time-dependent reduction of IκBα levels also in macrophages. LPS stimulation induced strong and sustained IκBα phosphorylation, while there was no detectable phosphorylation in cells treated with 5-ITu alone. LPS-induced degradation of IκBα was detectable at 30 min with a clear re-synthesis of this protein detectable at later time-points. Similar to the data for MEFs, 5-ITu strongly suppressed IκBα re-synthesis (Fig. 4D). Thus, 5-ITu seems to impair both TNF- and LPS-mediated signaling by interfering with the activity of the NFκB pathway.

In order to identify the direct target(s) of 5-ITu relevant to TNF-induced RIPK1 checkpoint signaling, we performed a differential scanning fluorimetry (DSF)-based selectivity kinome screen using 100 protein kinases. These kinases were selected based on their successful ectopic expression in E. coli and to represent a kinase panel that covers the entire kinome tree. The screen identified several kinase targets of 5-ITu, which showed a similar temperature shift upon 5-ITu binding comparable to the reference compound staurosporine, indicating strong inhibition. We identified about 20 kinases that displayed more than a 3-degree shift in Tm, with the top positions with more than 10 degrees shift occupied by CLK1 (13.3 degrees), GSG2/Haspin (12.3 degrees) and DYRK2 (10.8 degrees) (Supplementary Fig. 3A and Supplementary Table S3). A CLK1/4 inhibitor was also part of our original screen (TG003, Supplementary Table 2) and did not show any effect comparable to 5-ITu. Cell death assays performed in the presence of LDN192960 (DYRK2 and Haspin inhibitor) and T3-CLK (CLK1/2/3 inhibitor) also did not display any effect on TNF-induced cell death in the MK2-KO cells (data not shown). Therefore, further screens with extended kinase panels have to be conducted to identify relevant targets of 5-ITu.

## Discussion

It has been previously shown that MK2 limits RIPK1 activation by phosphorylating it at S320/S335 residues (S321/336 in murine RIPK1) in response to pro-inflammatory stimuli such as TNF, LPS and *Yersinia* infection, and therefore, can control RIPK1-dependent cell death [14–16]. Although previous reports did not specifically examine the mode of cell death resulting from loss of MK2, our data obtained in this study unequivocally demonstrate that even under pro-apoptotic conditions, MK2-deficient cells primarily undergo necroptosis. This is unexpected, since induction of necroptosis as a primary cytotoxic response to TNF/LPS in vitro usually requires CASP8 inhibition or FADD depletion. However, it has been established that CASP8 inhibition is not a prerequisite for necroptotic death in vivo [35]. Previously, proteasome inhibition was shown to induce necroptosis in MEFs and human leukemia cells without the need of caspase inhibition and TNF stimulation, but through the accumulation of K48-poly-ubiquitinated RIPK3 [36]. In this case, cell death was not RIPK1 activity-dependent, and RIPK3 inhibition switched it to apoptosis, as observed in the case of MK2 deficiency. It has also been recently shown that the accumulation of a critical amount of active MLKL directly at the plasma membrane is crucial for the execution of necroptosis [37]. Therefore, it is also possible that the level of active RIPK1 available to activate the necroptotic cascade is a crucial parameter for determining the cell death outcome. We further noted that inhibition of RIPK1 activity completely abrogated TNF-induced cell death in MK2-deficient cells treated with pro-apoptotic stimuli, while inhibition of RIPK3 activity predominantly resulted in apoptotic cell death. This is consistent with a previous study showing that inhibition of RIPK3 blocks necroptosis but induces apoptosis [38, 39]. Interestingly, expression of a RIPK1 deletion mutant lacking the intermediate domain which harbors the MK2 phosphorylation sites was shown to induce a shift from TNF-induced necroptosis to apoptosis in murine L929 cells [40]. The prominent caspase cleavage site on RIPK1 (D324) is also part of the intermediate domain and very close to the MK2 target site S320. The finding that mutations, which render RIPK1 caspase-resistant, are associated with autoinflammatory disease [41], further supports a role for the intermediate domain and the PTMs therein for cell fate and apoptosis/necroptosis decisions by RIPK1. Apart from this, loss of the p38/MK2 upstream kinase TAK1 was shown to be associated with increased sensitivity to TRAIL-induced necroptosis without the need for caspase inhibition [42]. This study revealed a novel connection between autophagosomes and necroptosis and showed RIPK1 activation and necrosome assembly templated by autophagosomes. In this case, depletion of components of the autophagy machinery induced the transition between necroptosis and apoptosis [42].

The screen for molecules that sensitize MK2-KO MEFs to TNF-induced necroptosis revealed 5-ITu as the most prominent candidate. The data indicated that 5-ITu, but not the related pan kinase inhibitors or adenosine kinase inhibitors, displayed this novel MK2 deficiency-associated phenotype of cell death. Moreover, we also verified a similar sensitizing effect of 5-ITu on the LPS-induced necroptotic response in macrophages. These findings indicated that 5-ITu was attenuating a RIPK1 activation checkpoint distinct from the MK2-dependent checkpoint, which has an additive effect on the inactivation of the p38/MK2 pathway. These findings are reminiscent to data on other RIPK1 checkpoint inhibitors of TBK1 and IKKs signaling. Consistently, we have observed a significant effect of 5-ITu in basal and TNF/LPS-induced NFκB signaling, which was mainly evident in cells with defective IκBα re-synthesis. While originally discovered as an adenosine kinase inhibitor with some inhibition on protein kinases CK1 and CK2, 5-ITu was later shown to strongly inhibit ERK2 [43]. Interestingly, a subsequent study used 5-ITu as an ERK1/2 inhibitor demonstrating inhibitory effects on silica-induced phosphorylation of p55 TNF receptor in RAW264.7 macrophages. In this study, 5-ITu also enhanced silica-induced macrophage apoptosis, a phenotype that was also promoted by dominant negative IκBα mutants [44]. Based on our findings, these data need to be re-evaluated to understand whether these effects can also be attributed to the 5-ITu-mediated inhibition of NFκB pathway. In addition to ERK2, Haspin/GSG2 is another well-characterized target of 5-ITu [26–28]. However, small-molecule inhibitors of Haspin and other potential 5-ITu targets identified in our DSF-based kinome screen also did not sensitize cells to TNF-induced death. This still leaves the explanation open that the phenotype of cell death and NFκB pathway inhibition shown by 5-ITu could be a combinatorial effect of inhibition of more than one protein kinase or a so far undiscovered target of 5-ITu. Interestingly, when we used PathwayNet tool [45] (https://pathwaynet.princeton.edu/) to understand functional interactions between the top candidates from the DSF-based screen (≥5 degree ∆*Tm*, *n* = 13), it revealed a high confidence, closely linked functional network between these protein kinases indicating a possible cooperation of these enzymes also in vivo (Supplementary Fig. 3B).

Cell death control in development, inflammation and infection is coordinated by RIPK1, but only a small part of the complex PTM code of RIPK1 is understood so far. In parallel to the first decoding of its PTMs, RIPK1-dependent necroptosis has become a sought-after target for positive and negative interference against cancer, neurodegeneration and inflammatory pathologies [46]. The identification of 5-ITu – an FDA-approved compound and genotoxic drug with anti-cancer potential—as modulator of RIPK1-dependent death, in combination with p38/MK2 inhibitors and SM opens possibilities for future therapies. Moreover, this adds 5-ITu to the list of anti-cancer agents such as Dabrafenib, Vemurafenib, Sorafenib, Pazopanib and Ponatinib, which are all multi-kinase targeting inhibitors and display therapeutic potential in diverse pathologies by interfering with the necroptosis pathway [47].

## Materials and methods

### Cell culture

SV40-T antigen immortalized WT, MK2- and MK2/3-deficient (DKO) MEFs and retroviral rescued cells were reported previously [48, 49]. MEFs and RAW 264.7 cells were cultured in DMEM supplemented with 10% fetal calf serum and 1% Penicillin/Streptomycin at 37 °C, in a humidified atmosphere supplemented with 5% CO_2_. Cells were routinely tested for mycoplasma by PCR.

### Antibodies and reagents

MLKL (#14993), Caspase-8 (#4927), p38 MAPK (#9212), MAPKAPK2 (D1E11) (#12155), pS166-RIPK1 (#31122), pS345-MLKL (#37333), phospho-p44/42 Erk1/2) (Thr202/Tyr204) (pERK1/2) (#4370), phospho-p38 (Thr180/Tyr182) (pp38) (#9215), phospho-SAPK/JNK (Thr183/Tyr185) (pJNK) (G9) (#9255), SAPK/JNK (#9252), pS32/36-IkBα (#9246), IkBα (#9242), pS176/180-IKKα/β (#2697), IKKα (#2682) antibodies were from Cell Signaling Technology (CST). Further antibodies used were against RIPK1 (#610459, BD Biosciences), GAPDH (#MAB374, Millipore), EF2 (sc-166415, Santa Cruz Biotechnology), ERK2 (sc-154, Santa Cruz Biotechnology). The secondary antibodies used were goat anti-mouse IgG (H+L)-HRP (#115-035-003, Dianova) and goat anti-rabbit IgG (H+L)-HRP (111-035-003, Dianova).

### Kinase inhibitors

The kinase inhibitor library, composed of 149 selective or broad-spectrum kinase inhibitors dissolved in DMSO (10 mM stock concentration), was purchased from Cayman Chemicals (#10505). The following reagents were used for cell treatments at given concentrations: LPS (*Escherichia* *coli* O55:B5, #L2880, Sigma-Aldrich, 100 ng ml^−1^), recombinant human TNFα (rHuTNF, #50435.50, Biomol, 10 ng/ml), Birinapant/SM (#HY-16591, MedChem Express, 1 µM), pan-caspase inhibitor zVAD-fmk (#4026865.0005, Bachem, 25 μM), MK2 inhibitor PF3644022 (#4279, Tocris, 5 μM), RIPK1 inhibitor Nec-1 (#BML-AP309-0020, Enzo Life Sciences, 50 µM), RIPK1 inhibitor Nec-1s (# 10-4544-5 mg, Tebu-Bio, 50 µM), RIPK3 inhibitor GSK872 (#HY-101872, MedChem Express, 5 µM), IKKβ inhibitor BMS345541 (#Axon 1731, Axon Medchem, 5 µM), TBK1/IKKε inhibitor BX795 (#T1830, Tebu-Bio), 5-Iodotubercidin (#HY-15424, MedChem Express, 2.5–10 µM), ABT-702 dihydrochloride (#HY-103161, MedChem Express, 5 µM), Staurosporine (#81590, Cayman, 5 µM), Etoposide (#E1383, Sigma, 5 µM), Doxorubicin (#15007, Cayman, 5 µM), Gemcitabine (#S1714, Selleckchem, 100 nM).

### Western blotting

Cells were grown in 12-well cell culture plates (60–70% confluency) and lysed directly in 200 µl of 2× SDS sample buffer containing 10% SDS, 1.5 M TRIS pH 8.8, 5% Glycerol, 2.5% 2-mercaptoethanol, and bromophenol blue, followed by denaturing the samples for 5 min at 95 °C. SDS-PAGE (7.5–16% gradient) gels were used for protein separation, followed by western blotting using nitrocellulose membrane (#10600001, Amersham Biosciences). Blotted membranes were blocked with 5% powdered skim milk in PBS with 0.1% Tween 20 for 1 h at room temperature. PBS with 0.1% Tween 20 was used for washing the membranes three times followed by incubation with the primary antibody overnight, at 4 °C. Next day, blots were washed and incubated with horseradish peroxidase-conjugated secondary antibodies for 1 h, at room temperature. ECL detection kit, WESTAR NOVA 2.0 (#XLS07105007, 7Biosciences) was used for the detection of proteins and the digital chemiluminescence images were taken by a Luminescent Image Analyser LAS-3000 (Fujifilm).

### Analysis of cell viability and cell death

CCK-8 assay (Bimake, #B334304) was used to measure cell viability. MEF cells and RAW264.7 cells were seeded in triplicates in a 96-well format in 100 µl media per well. Next day, cells were treated with various indicated combinations of reagents. For MEFs, the medium was changed with diluted WST-8 for viability estimation. For RAW 264.7 cells, 10 µl WST-8 was added directly to the wells in a complete medium. The plates were then incubated in a cell culture incubator for 1 h at 37 °C until the color turned orange and the absorbance was measured at 450 nm using a microplate reader (Perkin Elmer Wallac Victor2 1420 Multilabel Counter). Results are expressed as the percentage of cell viability per well in relation to the maximum cell viability of DMSO-treated cells. For the inhibitor screen, MEFs were seeded in triplicates and treated with 10 µM inhibitor samples of the panel, alone or in combination with 10 ng/ml TNF. For measuring cell death, the cell impermeable Sytox Green Nucleic Acid Stain (S7020, Invitrogen) was added to the samples after 30 min of TNF treatment, at a final concentration of 0.25 µM. The indicated treatments were performed in triplicates in a 96-well plate format. A microplate reader (Perkin Elmer Wallac Victor2 1420 Multilabel Counter) was used to measure the kinetics of fluorescence (excitation/emission filters: 485 nm/535 nm).

### DSF-based selectivity screening against a curated kinase library

The assay was performed as previously described [50, 51]. Briefly, recombinant protein kinase domains at a concentration of 2 μM were mixed with 10 μM compound in a buffer containing 20 mM HEPES, pH 7.5, and 500 mM NaCl. SYPRO Orange (5000×, Invitrogen) was added as a fluorescence probe (1 µl/ml). Subsequently, temperature-dependent protein unfolding profiles were measured using the QuantStudio™ 5 real-time PCR machine (Thermo Fisher). Excitation and emission filters were set to 465 and 590 nm, respectively. The temperature was raised with a step rate of 3 °C/min in the range of 25–85 °C. Data points were analyzed with the internal software (Thermal Shift Software^TM^ Version 1.4, Thermo Fisher) using the Boltzmann equation to determine the inflection point of the transition curve.

### Statistics and reproducibility

All immunoblot results presented in the figures are representative results from at least three independent experiments. All cell death and viability assays (except for the inhibitor screen) were performed in triplicate samples and the data presented are representative of three independent experiments. Inhibitor screen was performed with three biological replicates and the positive hits were reverified in an independent experiment. The calculations, statistical analyses and graphs were performed using Microsoft Excel. Two-tailed unpaired *t*-test was used to calculate the statistical significance of the viability assays. The statistics and source data are presented in Supplementary Table S4.

## Supplementary information

Legends to the Supplements

Suppl. Figures 1–3

Suppl. Table S1

Suppl. Table S2

Suppl. Table S3

Suppl. Table S4

Original Data File

## Data Availability

All data generated during this study leading to the findings presented here are included in this published article and its Supplementary data files.

## References

[1] Humphries F, Yang S, Wang B, Moynagh PN (2015). RIP kinases: key decision makers in cell death and innate immunity. Cell Death Differ.

[2] Varfolomeev E, Vucic D (2022). RIP1 post-translational modifications. Biochem J.

[3] Micheau O, Tschopp J (2003). Induction of TNF receptor I-mediated apoptosis via two sequential signaling complexes. Cell.

[4] Wang L, Du F, Wang X (2008). TNF-alpha induces two distinct caspase-8 activation pathways. Cell.

[5] Cho YS, Challa S, Moquin D, Genga R, Ray TD, Guildford M (2009). Phosphorylation-driven assembly of the RIP1-RIP3 complex regulates programmed necrosis and virus-induced inflammation. Cell.

[6] Sun L, Wang H, Wang Z, He S, Chen S, Liao D (2012). Mixed lineage kinase domain-like protein mediates necrosis signaling downstream of RIP3 kinase. Cell.

[7] Murphy JM, Czabotar PE, Hildebrand JM, Lucet IS, Zhang JG, Alvarez-Diaz S (2013). The pseudokinase MLKL mediates necroptosis via a molecular switch mechanism. Immunity.

[8] McComb S, Cessford E, Alturki NA, Joseph J, Shutinoski B, Startek JB, et al. Type-I interferon signaling through ISGF3 complex is required for sustained Rip3 activation and necroptosis in macrophages. Proc Natl Acad Sci USA. 2014;111:E3206–13. 10.1073/pnas.1407068111 PMC4128105 25049377

[9] Meylan E, Burns K, Hofmann K, Blancheteau V, Martinon F, Kelliher M (2004). RIP1 is an essential mediator of Toll-like receptor 3–induced NF-κB activation. Nat Immunol.

[10] He S, Liang Y, Shao F, Wang X (2011). Toll-like receptors activate programmed necrosis in macrophages through a receptor-interacting kinase-3-mediated pathway. Proc Natl Acad Sci USA.

[11] Tenev T, Bianchi K, Darding M, Broemer M, Langlais C, Wallberg F (2011). The Ripoptosome, a signaling platform that assembles in response to genotoxic stress and loss of IAPs. Mol Cell.

[12] Annibaldi A, Meier P (2018). Checkpoints in TNF-induced cell death: implications in inflammation and cancer. Trends Mol Med.

[13] Dondelinger Y, Jouan-Lanhouet S, Divert T, Theatre E, Bertin J, Gough PJ (2015). NF-κB-independent role of IKKα/IKKβ in preventing RIPK1 kinase-dependent apoptotic and necroptotic cell death during TNF signaling. Mol Cell.

[14] Menon MB, Gropengießer J, Fischer J, Novikova L, Deuretzbacher A, Lafera J (2017). p38MAPK/MK2-dependent phosphorylation controls cytotoxic RIPK1 signalling in inflammation and infection. Nat Cell Biol.

[15] Dondelinger Y, Delanghe T, Rojas-Rivera D, Priem D, Delvaeye T, Bruggeman I (2017). MK2 phosphorylation of RIPK1 regulates TNF-mediated cell death. Nat Cell Biol.

[16] Jaco I, Annibaldi A, Lalaoui N, Wilson R, Tenev T, Laurien L (2017). MK2 phosphorylates RIPK1 to prevent TNF-induced cell death. Mol Cell.

[17] Dondelinger Y, Delanghe T, Priem D, Wynosky-Dolfi MA, Sorobetea D, Rojas-Rivera D (2019). Serine 25 phosphorylation inhibits RIPK1 kinase-dependent cell death in models of infection and inflammation. Nat Commun.

[18] Lafont E, Draber P, Rieser E, Reichert M, Kupka S, de Miguel D (2018). TBK1 and IKKε prevent TNF-induced cell death by RIPK1 phosphorylation. Nat Cell Biol.

[19] Tu H, Xiong W, Zhang J, Zhao X, Lin X (2022). Tyrosine phosphorylation regulates RIPK1 activity to limit cell death and inflammation. Nat Commun.

[20] Du J, Xiang Y, Liu H, Liu S, Kumar A, Xing C (2021). RIPK1 dephosphorylation and kinase activation by PPP1R3G/PP1γ promote apoptosis and necroptosis. Nat Commun.

[21] Lalaoui N, Hänggi K, Brumatti G, Chau D, Nguyen NYN, Vasilikos L (2016). Targeting p38 or MK2 enhances the anti-leukemic activity of Smac-mimetics. Cancer Cell.

[22] Rijal D, Ariana A, Wight A, Kim K, Alturki NA, Aamir Z (2018). Differentiated macrophages acquire a pro-inflammatory and cell death-resistant phenotype due to increasing XIAP and p38-mediated inhibition of RipK1. J Biol Chem.

[23] Davies LP, Jamieson DD, Baird-Lambert JA, Kazlauskas R (1984). Halogenated pyrrolopyrimidine analogues of adenosine from marine organisms: pharmacological activities and potent inhibition of adenosine kinase. Biochem Pharmacol.

[24] Massillon D, Stalmans W, Van de Werve G, Bollen M (1994). Identification of the glycogenic compound 5-iodotubercidin as a general protein kinase inhibitor. Biochem J.

[25] Dirice E, Walpita D, Vetere A, Meier BC, Kahraman S, Hu J (2016). Inhibition of DYRK1A stimulates human β-cell proliferation. Diabetes.

[26] De Antoni A, Maffini S, Knapp S, Musacchio A, Santaguida S (2012). A small-molecule inhibitor of Haspin alters the kinetochore functions of Aurora B. J Cell Biol.

[27] Karanika E, Soupsana K, Christogianni A, Stellas D, Klinakis A, Politou AS (2020). Haspin-dependent and independent effects of the kinase inhibitor 5-Iodotubercidin on self-renewal and differentiation. Sci Rep.

[28] Heroven C, Georgi V, Ganotra GK, Brennan P, Wolfreys F, Wade RC (2018). Halogen-aromatic π interactions modulate inhibitor residence times. Angew Chem Int Ed Engl.

[29] Zhang X, Jia D, Liu H, Zhu N, Zhang W, Feng J (2013). Identification of 5-Iodotubercidin as a genotoxic drug with anti-cancer potential. PLoS ONE.

[30] Zhao J, Liu Q, Yi D, Li Q, Guo SS, Ma L (2022). 5-Iodotubercidin inhibits SARS-CoV-2 RNA synthesis. Antivir Res.

[31] Wang Q, Fan D, Xia Y, Ye Q, Xi X, Zhang G, et al. The latest information on the RIPK1 post-translational modifications and functions. Biomed Pharmacother. 2021;142:112082. 10.1016/j.biopha.2021.112082 34449307

[32] Parkinson FE, Geiger JD (1996). Effects of iodotubercidin on adenosine kinase activity and nucleoside transport in DDT1 MF-2 smooth muscle cells. J Pharmacol Exp Ther.

[33] Ugarkar BG, DaRe JM, Kopcho JJ, Browne CE, Schanzer JM, Wiesner JB (2000). Adenosine kinase inhibitors. 1. Synthesis, enzyme inhibition, and antiseizure activity of 5-iodotubercidin analogues. J Med Chem.

[34] Radek RJ, Decker MW, Jarvis MF (2004). The adenosine kinase inhibitor ABT-702 augments EEG slow waves in rats. Brain Res.

[35] Duprez L, Takahashi N, Van Hauwermeiren F, Vandendriessche B, Goossens V, Vanden Berghe T (2011). RIP kinase-dependent necrosis drives lethal systemic inflammatory response syndrome. Immunity.

[36] Moriwaki K, Chan FKM (2016). Regulation of RIPK3- and RHIM-dependent necroptosis by the proteasome. J Biol Chem.

[37] Samson AL, Zhang Y, Geoghegan ND, Gavin XJ, Davies KA, Mlodzianoski MJ (2020). MLKL trafficking and accumulation at the plasma membrane control the kinetics and threshold for necroptosis. Nat Commun.

[38] Mandal P, Berger SB, Pillay S, Moriwaki K, Huang C, Guo H (2014). RIP3 induces apoptosis independent of pronecrotic kinase activity. Mol Cell.

[39] Newton K, Dugger DL, Maltzman A, Greve JM, Hedehus M, Martin-McNulty B (2016). RIPK3 deficiency or catalytically inactive RIPK1 provides greater benefit than MLKL deficiency in mouse models of inflammation and tissue injury. Cell Death Differ.

[40] Duprez L, Bertrand MJM, Berghe TV, Dondelinger Y, Festjens N, Vandenabeele P (2012). Intermediate domain of receptor-interacting protein kinase 1 (RIPK1) determines switch between necroptosis and RIPK1 kinase-dependent apoptosis. J Biol Chem.

[41] Lalaoui N, Boyden SE, Oda H, Wood GM, Stone DL, Chau D (2020). Mutations that prevent caspase cleavage of RIPK1 cause autoinflammatory disease. Nature.

[42] Goodall ML, Fitzwalter BE, Zahedi S, Wu M, Rodriguez D, Mulcahy-Levy JM (2016). The autophagy machinery controls cell death switching between apoptosis and necroptosis. Dev Cell.

[43] Fox T, Coll JT, Xie X, Ford PJ, Germann UA, Porter MD (1998). A single amino acid substitution makes ERK2 susceptible to pyridinyl imidazole inhibitors of p38 MAP kinase. Protein Sci.

[44] Gambelli F, Di P, Niu X, Friedman M, Hammond T, Riches DWH (2004). Phosphorylation of tumor necrosis factor receptor 1 (p55) protects macrophages from silica-induced apoptosis. J Biol Chem.

[45] Park CY, Krishnan A, Zhu Q, Wong AK, Lee YS, Troyanskaya OG (2015). Tissue-aware data integration approach for the inference of pathway interactions in metazoan organisms. Bioinformatics.

[46] Degterev A, Ofengeim D, Yuan J (2019). Targeting RIPK1 for the treatment of human diseases. Proc Natl Acad Sci USA.

[47] Fulda S (2018). Repurposing anticancer drugs for targeting necroptosis. Cell Cycle.

[48] Ronkina N, Kotlyarov A, Dittrich-Breiholz O, Kracht M, Hitti E, Milarski K (2007). The mitogen-activated protein kinase (MAPK)-activated protein kinases MK2 and MK3 cooperate in stimulation of tumor necrosis factor biosynthesis and stabilization of p38 MAPK. Mol Cell Biol.

[49] Ronkina N, Menon MB, Schwermann J, Arthur JSC, Legault H, Telliez JB (2011). Stress induced gene expression: a direct role for MAPKAP kinases in transcriptional activation of immediate early genes. Nucleic Acids Res.

[50] Krämer A, Kurz CG, Berger BT, Celik IE, Tjaden A, Greco FA (2020). Optimization of pyrazolo[1,5-a]pyrimidines lead to the identification of a highly selective casein kinase 2 inhibitor. Eur J Med Chem.

[51] Fedorov O, Niesen FH, Knapp S (2012). Kinase inhibitor selectivity profiling using differential scanning fluorimetry. Methods Mol Biol.

