# Supplementary material for: 5-Iodotubercidin sensitizes cells to RIPK1-dependent necroptosis by interfering with NFκB signaling
Source: Cell Death Discov. 2023 Jul 26;9:262. doi: 10.1038/s41420-023-01576-x (PMC10372004; doi:10.1038/s41420-023-01576-x)
Supplement: Supplementary file 3 — Suppl. Table S1 [file 41420_2023_1576_MOESM3_ESM.pdf]

Supplementary Table 1. Small molecule library used for cell viability screen

Sl No.	Product Name	Description	Synonyms	CAS Number	Formula Weight	Molecular Formula	Target
1	TG003	Potent inhibitor of CDC 2-like kinase		300801-52-9	249,3	$C_{13}H_{15}NO_2S$	CLK1, CLK4
2	PKC 412	Kinase inhibitor with potential use in cancer treatment	N-Benzoylstaurosporine CGP 41231 CGP 41251 Midostaurin	120685-11-2	570,6	$C_{35}H_{30}N_4O_4$	Multiple Kinases
3	Doramapimod	A potent inhibitor of p38 MAPK	BIRB-796	285983-48-4	527,7	$C_{31}H_{37}N_5O_3$	p38 MAPK
4	Paclitaxel	A potent mitotic inhibitor	NSC 125973	33069-62-4	853,9	$C_{47}H_{51}NO_{14}$	Tubulin
5	Erlotinib	An EGFR tyrosine kinase inhibitor	NSC 718781	183321-74-6	393,4	$C_{22}H_{23}N_3O_4$	EGFR
6	NVP-BEZ235	A dual inhibitor of class I PI3Ks and mTOR	Dactolisib	915019-65-7	469,5	$C_{30}H_{23}N_5O$	
7	Phthalazinone pyrazole	Potent, selective inhibitor of Aurora kinase A		880487-62-7	317,4	$C_{18}H_{15}N_5O$	Aurora A
8	AG-879	A non-specific tyrphostin ErbB2 inhibitor	Tyrphostin AG-879	148741-30-4	316,5	$C_{18}H_{24}N_2OS$	BMX
9	1-NA-PP1	Inhibitor of modified 'analog-sensitive' kinases	1-Naphthyl-PP1 PP1 Analog	221243-82-9	317,4	$C_{19}H_{19}N_5$	
10	Torin 1	Selective inhibitor of mTOR		1222998-36-8	607,6	$C_{35}H_{28}F_3N_5O_2$	mTORC1, mTORC2
11	BIBF 1120	A VEGFR, FGFR, and PDGFR inhibitor	Nintedanib	656247-17-5	539,6	$C_{31}H_{33}N_5O_4$	FGFR, PDGFR, VEGFR
12	SMI-4a	A Pim kinase inhibitor		438190-29-5	273,2	$C_{11}H_6F_3NO_2S$	PIMs
13	CAY10657	A likely inhibitor of IKK2 kinase		494772-86-0	360,4	$C_{17}H_{20}N_4O_3S$	
14	Chelerythrine (chloride)	Potent inhibitor of PKC and Bcl-xL	Broussonpapyrine chloride NSC 646662	3895-92-9	383,8	$C_{21}H_{18}NO_4 \cdot Cl$	Bcl-xL PKC
15	Tunicamycin Mixture	An antimicrobial and inhibitor of glycosylation		11089-65-9	845,0	$C_{39}H_{64}N_4O_{16}$ (for Tunicamycin VII)	
16	AZD 7762	A selective checkpoint kinase inhibitor		860352-01-8	362,4	$C_{17}H_{19}FN_4O_2S$	Chk1, Chk2
17	GSK1059615	A potent PI3K inhibitor		958852-01-2	333,4	$C_{18}H_{11}N_3O_2S$	PI3Kα
18	Ruxolitinib	A potent, selective JAK1/JAK2 inhibitor	INCB 018424	941678-49-5	306,4	$C_{17}H_{18}N_6$	JAK1, JAK2
19	Necrostatin-1	A RIP1 kinase inhibitor	Nec-1	4311-88-0	259,3	$C_{13}H_{13}N_3OS$	RIPK1
20	SB-505124	Inhibitor of receptors ALK4, ALK5, and ALK7		694433-59-5	335,4	$C_{20}H_{21}N_3O_2$	
21	INK128	Inhibitor of TORC1/2	MLN0128	1224844-38-5	309,3	$C_{15}H_{15}N_7O$	mTOR
22	Canertinib (hydrochloride)	A pan-ErbB tyrosine kinase inhibitor	CI-1033 PD 183805	289499-45-2	558,9	$C_{24}H_{25}ClFN_5O_3 \cdot {}_2HCl$	EGFR
23	SB-431542 (hydrate)	Inhibitor of receptors ALK4, ALK5, and ALK7			384,4	$C_{22}H_{16}N_4O_3 \cdot XH_2O$	ALK5
24	PD 173074	Inhibitor of tyrosine kinase activity of fibroblast growth factor receptors		219580-11-7	523,7	$C_{28}H_{41}N_7O_3$	FGFR1
25	Valproic Acid (sodium salt)	A class I HDAC inhibitor	2-Propylvaleric Acid Valproate VPA	1069-66-5	166,2	$C_8H_{15}O_2 \cdot Na$	HDAC
26	PD 0325901	A MEK inhibitor that sustains stem cell renewal		391210-10-9	482,2	$C_{16}H_{14}F_3IN_2O_4$	MEK
27	SB 203580	A specific p38 MAPK inhibitor	PB 203580 RWJ 64809	152121-47-6	377,4	$C_{21}H_{16}FN_3OS$	p38 MAPK
28	VX-702	An inhibitor of p38 MAP kinases		745833-23-2	404,3	$C_{19}H_{12}F_4N_4O_2$	p38 MAPK
29	Emodin	Natural CK2 inhibitor and ER agonist	Archin Frangulic Acid NSC 408120 NSC 622947 Schuttgelb	518-82-1	270,2	$C_{15}H_{10}O_5$	CK2
30	CHIR99021	A selective GSK3 inhibitor	CT 99021	252917-06-9	465,3	$C_{22}H_{18}Cl_2N_8$	GSK3α, GSK3β
31	BIO	A potent, selective, and reversible GSK3 inhibitor	6-Bromoindirubin-3'-oxime GSK3 Inhibitor IX MLS 2052	667463-62-9	356,2	$C_{16}H_{10}BrN_3O_2$	GSK3α, GSK3β
32	Imatinib (mesylate)	An inhibitor of c-Abl, Bcr-Abl, PDGFR, and c-Kit	CGP57148B STI-571	220127-57-1	589,7	$C_{29}H_{31}N_7O \cdot CH_4SO_3$	Multiple Kinases
33	Sunitinib (malate)	A multi-kinase inhibitor	SU11248	341031-54-7	532,6	$C_{22}H_{27}FN_4O_2 \cdot C_4H_6O_5$	FLK1, FLT3, PDGFRβ
34	Gefitinib	An EGFR inhibitor	ZD 1839	184475-35-2	446,9	$C_{22}H_{24}ClFN_4O_3$	
35	PP2	A selective inhibitor of Src tyrosine kinases	AGL 1879	172889-27-9	301,8	$C_{15}H_{16}ClN_5$	Fyn, HCK, LYN
36	3-Methyladenine	An inhibitor of autophagy	3-MA NSC 66389	5142-23-4	149,2	$C_6H_7N_5$	PI3K
37	Bisindolylmaleimide I	A PKC inhibitor	BIM I GF 109203X Gö 6850	133052-90-1	412,5	$C_{25}H_{24}N_4O_2$	PKC
38	Bisindolylmaleimide IV	A PKC inhibitor	Arcyriarubin A BIM IV	119139-23-0	327,3	$C_{20}H_{13}N_3O_2$	
39	Bisindolylmaleimide V	A S6K inhibitor	BIM V Ro 31-6045	113963-68-1	341,3	$C_{21}H_{15}N_3O_2$	S6K
40	NSC 663284	Inhibitor of Cdc25 isoforms	Cdc25 Phosphatase Inhibitor II DA-3003-1 SPS8I1	383907-43-5	321,8	$C_{15}H_{16}ClN_3O_3$	
41	D 4476	Inhibitor of CK1 and ALK5	Casein Kinase I Inhibitor	301836-43-1	398,4	$C_{23}H_{18}N_4O_3$	CK1
42	NU 7026	Inhibitor of DNA-dependent protein kinase	DNA-PK Inhibitor II LY293646	154447-35-5	281,3	$C_{17}H_{15}NO_3$	DNA-PK
43	Gö 6983	Inhibitor of protein kinase C		133053-19-7	442,5	$C_{26}H_{26}N_4O_3$	PKC
44	H-9 (hydrochloride)	A potent, nonspecific kinase inhibitor	Protein Kinase Inhibitor H-9	116700-36-8	324,2	$C_{11}H_{13}N_3O_2S \cdot {}_2HCl$	PKG
45	Indirubin-3'-monoxime	Inhibitor of GSK3β and cyclin-dependent kinases		160807-49-8	277,3	$C_{16}H_{11}N_3O_2$	GSK3β
46	NU 6102	A potent Cdk1 and Cdk2 inhibitor		444722-95-6	402,5	$C_{18}H_{22}N_6O_3S$	Cdk1, Cdk2
47	KN-62	Inhibitor of Ca2+/calmodulin-dependent kinase type II		127191-97-3	721,9	$C_{38}H_{35}N_5O_6S_2$	CAMKII
48	KN-93	Selective inhibitor of Ca2+/calmodulin-dependent kinase type II		139298-40-1	501,0	$C_{26}H_{29}ClN_2O_4S$	CAMKII
49	CGP 57380	Inhibitor of MAPK-interacting kinase1	MNK1 Inhibitor	522629-08-9	244,2	$C_{11}H_9FN_6$	MNK1
50	Iso-Olomoucine	An inactive stereoisomer of olomoucine		101622-50-8	298,3	$C_{15}H_{18}N_6O$	
51	(S)-Glycyl-H-1152 (hydrochloride)	A ROCK inhibitor	Rho Kinase Inhibitor IV	913844-45-8	449,4	$C_{18}H_{24}N_4O_3S \cdot {}_2HCl$	ROCK-II
52	Bisindolylmaleimide VIII (acetate)	A PKC inhibitor	BIM VIII Ro 31-7549	138516-31-1	458,5	$C_{24}H_{22}N_4O_2 \cdot C_2H_4O_2$	
53	Bisindolylmaleimide IX (mesylate)	Inhibitor of protein kinase C	BIM IX Ro 31-8220	138489-18-6	553,7	$C_{25}H_{23}N_5O_2S \cdot CH_3SO_3H$	GSK3 PKC
54	ST638	Tyrosine kinase inhibitor		107761-24-0	354,4	$C_{19}H_{18}N_2O_3S$	
55	SU6656	Inhibitor of Src kinases		330161-87-0	371,5	$C_{19}H_{21}N_3O_3S$	Yes
56	LY364947	Inhibitor of TGF-β type-1 receptors	HTS 466284 TGF-β RI Kinase Inhibitor	396129-53-6	272,3	$C_{17}H_{12}N_4$	ALK5
57	SB 203580 (hydrochloride)	A water soluble p38 MAPK inhibitor	PB 203580 RWJ 64809	869185-85-3	413,9	$C_{21}H_{16}FN_3OS \cdot HCl$	
58	CAY10621	Selective inhibitor of SPHK1	SKI 5C SPHK1 Inhibitor 5C	120005-55-2	435,6	$C_{26}H_{45}NO_4$	SPHK1
59	YM-201636	Inhibitor of PIKfyve		371942-69-7	467,5	$C_{25}H_{21}N_7O_3$	PIKfyve
60	ZM 447439	Selective inhibitor of Aurora B kinase		331771-20-1	513,6	$C_{29}H_{31}N_5O_4$	Aurora B
61	AS-041164	Selective inhibitor of PI3Kγ		6318-41-8	249,2	$C_{11}H_7NO_4S$	PI3Kγ
62	NVP-AEW541 (hydrochloride)	An IGF-IR antagonist		2320261-63-8	512,5	$C_{27}H_{29}N_5O \cdot {}_2HCl$	IGF-1R
63	PP242	Potent inhibitor of mTOR kinase in both mTORC1 and mTORC2		1092351-67-1	308,3	$C_{16}H_{16}N_6O$	mTOR
64	ABT-869	An effective inhibitor of VEGF and PDGF receptor kinases	Linifanib	796967-16-3	375,4	$C_{21}H_{18}FN_5O$	PDGFR family, VEGFR family
65	CAY10622	A ROCK1 and ROCK2 inhibitor		1038549-25-5	443,5	$C_{25}H_{25}N_5O_3$	ROCK-I ROCK-II
66	17β-hydroxy Wortmannin	Inhibitor of phosphoinoside 3-kinase		58053-83-1	430,4	$C_{23}H_{26}O_8$	PI3K
67	CAY10626	A dual PI3Kα/mTOR kinase inhibitor		1202884-94-3	624,7	$C_{31}H_{35}F_3N_8O_3$	mTOR, PI3Kα
68	SU 6668	An inhibitor of select receptor tyrosine kinases	NSC 702827 Orantinib TSU-68	252916-29-3	310,4	$C_{18}H_{18}N_2O_3$	Aurora B
69	PHA-767491 (hydrochloride)	A potent Cdc7 kinase inhibitor	CAY10572	942425-68-5	249,7	$C_{12}H_{11}N_3O \cdot HCl$	Cdc7
70	N,N-Dimethylsphingosine (d18:1)	A SPHK inhibitor		119567-63-4	327,6	$C_{20}H_{41}NO_2$	
71	LY294002	A selective PI3K inhibitor		154447-36-6	307,3	$C_{19}H_{17}NO_3$	PI3K
72	U-0126	A MEK inhibitor and AMPK activator		109511-58-2	380,5	$C_{18}H_{16}N_6S_2$	MEK
73	Staurosporine	A potent inhibitor of protein kinase C	Stsp	62996-74-1	466,5	$C_{28}H_{26}N_4O_3$	PKCα
74	AS-605240 (potassium salt)	A potent inhibitor of PI3Kγ			295,4	$C_{12}H_6N_3O_2S \cdot K$	

75	PD 166326	An inhibitor of c-src and certain receptor tyrosine kinases, including c-abl		185039-91-2	427,3	$C_{21}H_{16}Cl_2N_4O_2$	
76	O-1918	A selective antagonist of abnormal cannabidiol-mediated effects		536697-79-7	286,4	$C_{19}H_{26}O_2$	
77	Y-27632 (hydrochloride)	A ROCK inhibitor		129830-38-2	320,3	$C_{14}H_{21}N_3O \cdot 2HCl$	PRK2, ROCK1, ROCK2,
78	Leelamine	An inhibitor of pyruvate dehydrogenase kinase	Dehydroabietylamine NSC 2955	1446-61-3	285,5	$C_{20}H_{31}N$	PDK
79	PD 98059	A MAPK pathway inhibitor and AMPK activator	NSC 679828	167869-21-8	267,3	$C_{16}H_{13}NO_3$	
80	PD 169316	A specific p38 MAPK inhibitor		152121-53-4	360,3	$C_{20}H_{13}FN_4O_2$	p38 MAPK
81	TGX-221	A potent, selective PI3K inhibitor		663619-89-4	364,4	$C_{21}H_{24}N_4O_2$	PI3K p110β
82	(S)-H-1152 (hydrochloride)	A ROCK inhibitor		451462-58-1	392,3	$C_{16}H_{21}N_3O_2S \cdot 2HCl$	ROCK
83	AS-605240	A potent inhibitor of PI3-kinase γ		648450-29-7	257,3	$C_{12}H_7N_3O_2S$	PI3Kα,β,δ,γ PI3Kγ
84	Sphingosine (d18:1)	A pharmacological tool to probe the activity of protein kinase C	(-)-Sphingosine D-erythro-Sphingosine C-18	123-78-4	299,5	$C_{18}H_{37}NO_2$	
85	JNJ-10198409	A potent PDGF tyrosine kinase inhibitor		627518-40-5	325,3	$C_{18}H_{16}FN_3O_2$	PDGFR
86	Leelamine (hydrochloride)	An inhibitor of pyruvate dehydrogenase kinase	Dehydroabietylamine	16496-99-4	321,9	$C_{20}H_{31}N \cdot HCl$	
87	Lauric Acid Leelamide	A novel fatty acid amide			467,8	$C_{32}H_{53}NO$	
88	AS-252424	A potent, selective inhibitor of PI3-kinase γ		900515-16-4	305,3	$C_{14}H_8FNO_4S$	
89	CAY10505	Inhibitor of PI3Kγ		328960-84-5	289,3	$C_{14}H_8FNO_3S$	CK2
90	PI-103	A potent, cell-permeable PI3-kinase inhibitor		371935-74-9	348,4	$C_{19}H_{16}N_4O_3$	DNA-PK, mTOR, PI3K
91	PIK-75 (hydrochloride)	A selective p110α inhibitor		372196-77-5	488,7	$C_{16}H_{14}BrN_5O_4S \cdot HCl$	PI3K p110α
92	Sphingosine Kinase Inhibitor 2	An SPHK1 inhibitor	SKI II SPHK I2	312636-16-1	302,8	$C_{15}H_{11}ClN_2OS$	SPHK1
93	Piceatannol	A potent resveratrol analog	Astringenin trans-Picetannol trans-3,3',4,5'-Tetrahydroxystilbene	10083-24-6	244,2	$C_{14}H_{12}O_4$	
94	SC-1	A synthetic compound that promotes self-renewal of murine embryonic stem cells	Pluripotin	839707-37-8	550,5	$C_{27}H_{25}F_3N_8O_2$	Stem Cell Renewal
95	(R)-Roscovitine	A potent inhibitor of cyclin-dependent kinase 2	Seliciclib	186692-46-6	354,5	$C_{19}H_{26}N_6O$	CDKs
96	Sorafenib	A multi-kinase inhibitor	BAY 43-9006	284461-73-0	464,8	$C_{21}H_{16}ClF_3N_4O_3$	pan-Kinase System xc- Cystine-glutamate Transporter
97	CAY10561	A selective inhibitor of ERK	Pyrazolylpyrrole ERK Inhibitor	933786-58-4	459,3	$C_{22}H_{17}Cl_2FN_4O_2$	ERK2
98	AS-604850	A selective inhibitor of PI3Kγ		648449-76-7	285,2	$C_{11}H_5F_2NO_4S$	
99	PI3-Kinase α Inhibitor 2	A PI3K p110α inhibitor	PI3Kα Inhibitor 2 Phosphatidylinositol 3-Kinase α Inhibitor 2	371943-05-4	313,4	$C_{16}H_{15}N_3O_2S$	PI3Kα
100	CAY10567	An Akt1 translocation inhibitor	BML-257	32387-96-5	326,4	$C_{21}H_{14}N_2O_2$	
101	ML-9	A PKB/Akt inhibitor		4870	361,3	$C_{15}H_{17}ClN_2O_2S \cdot HCl$	Multiple Kinases
102	Triciribine	A selective, potent inhibitor of Akt activation	API 2 NSC 154020 Tricyclic Nucleoside	35943-35-2	320,3	$C_{13}H_{16}N_6O_4$	
103	Erbstatin analog	An EGFR tyrosine kinase inhibitor	Methyl 2,5-dihydoxycinnamate	63177-57-1	194,2	$C_{10}H_{10}O_4$	EGFR
104	Kenpaullone	A inhibitor of cyclin-dependent kinase and GSK3β	9-Bromopaullone NSC 664704	142273-20-9	327,2	$C_{16}H_{11}BrN_2O$	CDKs, GSK3β
105	Olomoucine	An inhibitor of cyclin-dependent kinases		101622-51-9	298,3	$C_{15}H_{18}N_6O$	CDKs
106	AG-494	An inhibitor of EGF receptor kinase	Tyrphostin AG-494	133550-35-3	280,3	$C_{16}H_{12}N_2O_3$	EGFR
107	AG-825	An inhibitor of Her2/Neu tyrosine kinase activity	Tyrphostin AG-825	149092-50-2	397,5	$C_{19}H_{15}N_3O_3S_2$	ErbB2
108	AG-1478	An inhibitor of EGF receptor kinase	NSC 693255 Tyrphostin AG-1478	153436-53-4	315,8	$C_{16}H_{14}ClN_3O_2$	EGFR
109	SB-216763	An inhibitor of GSK3		280744-09-4	371,2	$C_{19}H_{12}Cl_2N_2O_2$	GSK3
110	SB-415286	A selective inhibitor of GSK-3		264218-23-7	359,7	$C_{16}H_{10}ClN_3O_5$	
111	AG-17	An inhibitor of EGF receptor kinase	GCP 5126 Malonoben NSC 242557 RG-50872 SF 6847 Tyrphostin 9 Tyrphostin AG-17	10537-47-0	282,4	$C_{18}H_{22}N_2O$	
112	H-8 (hydrochloride)	A potent, nonspecific kinase inhibitor	Protein Kinase Inhibitor H-8	113276-94-1	338,3	$C_{12}H_{15}N_3O_2S \cdot 2HCl$	PKA PKG
113	LFM-A13	A BTK inhibitor		244240-24-2	360,0	$C_{11}H_8Br_2N_2O_2$	BTK
114	SC-514	Selective inhibitor of IKK2	GK 01140	354812-17-2	224,3	$C_9H_8N_2OS_2$	IKKβ
115	Apigenin	A CK2 inhibitor	Chamomile Flavone NSC 83244 Versulin	520-36-5	270,2	$C_{15}H_{10}O_5$	
116	AG-18	An inhibitor of EGF receptor kinase	RG-50810 RG-50858 TX 825 Tyrphostin 23 Tyrphostin AG-18	118409-57-7	186,1	$C_{10}H_6N_2O_2$	
117	DRB	A CTD kinase inhibitor	Benzimidazole NSC 401575	53-85-0	319,1	$C_{12}H_{12}Cl_2N_2O_4$	
118	RG-13022	An inhibitor of EGF receptor kinase		149286-90-8	266,3	$C_{16}H_{14}N_2O_2$	EGFR
119	RG-14620	An inhibitor of EGF receptor kinase		136831-49-7	275,1	$C_{14}H_8Cl_2N_2$	
120	AG-490	An inhibitor of protein tyrosine kinase	Tyrphostin AG-490	133550-30-8	294,3	$C_{17}H_{14}N_2O_3$	JAK2
121	AG-82	An inhibitor of EGF receptor kinase	NSC 676484 RG-50875 Tyrphostin 25 Tyrphostin AG-82	118409-58-8	202,2	$C_{10}H_6N_2O_3$	
122	AG-99	An inhibitor of EGF receptor kinase	Tyrphostin 46 Tyrphostin AG-99	122520-85-8	204,2	$C_{10}H_8N_2O_3$	
123	AG-213	An inhibitor of EGF receptor kinase	Tyrphostin AG-213 Tyrphostin 47	122520-86-9	220,2	$C_{10}H_8N_2O_2S$	
124	AG-183	An inhibitor of EGF receptor kinase	Tyrphostin 51	122520-90-5	268,2	$C_{13}H_8N_4O_3$	EGFR
125	Lavendustin C	A potent tyrosine kinase inhibitor	HDBA NSC 666251	125697-93-0	275,3	$C_{14}H_{13}NO_5$	EGFR
126	5-Iodotubercidin	A protein kinase inhibitor	Itu NSC 113939	24386-93-4	392,2	$C_{11}H_{13}IN_4O_4$	CK1, ERK2, PKC
127	SB 202190	A specific and potent p38 MAP kinase inhibitor		152121-30-7	331,3	$C_{20}H_{14}FN_3O$	p38 MAPK
128	CAY10571	A potent anti-inflammatory agent		152121-46-5	393,4	$C_{21}H_{16}FN_3O_2S$	p38α MAPK
129	Nilotinib	A tyrosine kinase inhibitor	AMN107	641571-10-0	529,5	$C_{28}H_{22}F_3N_7O$	Bcr-Abl
130	SP 600125	A pan-JNK inhibitor	NSC 75890 1PMV Pyrazolanthrone	129-56-6	220,2	$C_{14}H_8N_2O$	JNKs
131	L-threo-Sphingosine (d18:1)	A D-erythro-sphingosine analog	L-threo-Sphingosine C18	25695-95-8	299,5	$C_{18}H_{37}NO_2$	
132	H-89 (hydrochloride)	A PKA inhibitor	5-Isoquinolinesulfonamide Protein Kinase Inhibitor H-89	130964-39-5	519,3	$C_{20}H_{20}BrN_3O_2S \cdot 2HCl$	Multiple Kinases
133	HA-1077 (hydrochloride)	A ROCK inhibitor	Fasudil	203911-27-7	364,3	$C_{14}H_{17}N_3O_2S \cdot 2HCl$	ROCK-II
134	AG-370	A selective inhibitor of PDGF receptor kinase.	NSC 651712	134036-53-6	259,3	$C_{15}H_9N_5$	PDGFR
135	Wortmannin	An irreversible PI3K inhibitor	KY 12420	19545-26-7	428,4	$C_{23}H_{24}O_8$	PI3K, Plk1, Plk3
136	AG-1296	An inhibitor of PDGF receptor kinase	Tyrphostin AG-1296	146535-11-7	266,3	$C_{16}H_{14}N_2O_2$	PDGFR
137	WHI-P131	A selective JAK3 inhibitor	Janex 1	202475-60-3	297,3	$C_{16}H_{15}N_3O_3$	JAK3
138	CAY10574	A Cdk2-cyclin E and Cdk9 inhibitor		140651-18-9	218,2	$C_9H_{10}N_6O$	Cdk9
139	CAY10576	A potent and selective inhibitor of IKKε		862812-98-4	469,5	$C_{22}H_{19}N_3O_5S_2$	IKKε
140	TWS119	A GSK3β inhibitor		601514-19-6	318,3	$C_{18}H_{14}N_4O_2$	GSK3β
141	NSC 210902	A selective CK2 inhibitor		51726-83-1	239,2	$C_{14}H_9NO_3$	
142	CAY10577	A CK2 inhibitor		300675-28-9	258,1	$C_{10}H_5Cl_2NO_3$	
143	CAY10578	A potent and selective CK2 inhibitor		19231-60-8	708,8	$C_{10}H_3I_4NO_4$	CK2
144	PD 184161	A potent MEK1/2 inhibitor		212631-67-9	557,6	$C_{17}H_{13}BrClF_2IN_2O_2$	MEK1, MEK2
145	CCT018159	An Hsp90 inhibitor		171009-07-7	352,4	$C_{20}H_{20}N_2O_4$	
146	Myricetin	A potent antioxidant	Cannabiscetin LDN-0014058 NSC 407290	529-44-2	318,2	$C_{15}H_{10}O_8$	
147	Necrostatin-5	A RIP1 kinase inhibitor	Nec-5	337349-54-9	383,5	$C_{19}H_{17}N_3O_2S_2$	RIPK1
148	OSU03012	An anti-cancer celecoxib analog		742112-33-0	460,5	$C_{26}H_{19}F_3N_4O$	
149	CAY10554	A potent inhibitor of Cdk5 and Cdk2	BML-259	267654-00-2	260,4	$C_{14}H_{16}N_2OS$	
