# Supplementary material for: 5-Iodotubercidin sensitizes cells to RIPK1-dependent necroptosis by interfering with NFκB signaling
Source: Cell Death Discov. 2023 Jul 26;9:262. doi: 10.1038/s41420-023-01576-x (PMC10372004; doi:10.1038/s41420-023-01576-x)
Supplement: Supplementary file 6 — Suppl. Table S4 [file 41420_2023_1576_MOESM6_ESM.pdf]

Supplementary Table S4: Statistics and source data for the quantitative results.

Figure 3A

Representative experiment

		Control	2,5µM 5-ITu	5µM 5-ITu	10µM 5-ITu	2,5µM 5-ITu+TNF	5µM 5-ITu+TNF	10µM 5-ITu+TNF	
MK2 KO+ MK2	Biol. Replicate 1	95,31	95,28	89,41	87,18	74,77	75,59	65,72	
	Biol. Replicate 2	100,43	93,30	85,11	81,32	75,72	71,80	66,56	
	Biol. Replicate 3	104,27	89,22	84,78	78,88	78,21	73,54	70,75	
MK2 KO+ vector	Biol. Replicate 1	97,67	88,17	76,83	84,11	43,05	32,26	24,38	
	Biol. Replicate 2	100,72	85,12	80,51	82,65	43,87	30,85	24,77	
	Biol. Replicate 3	101,61	87,83	79,65	76,90	44,21	28,17	24,67	
p-values (MK2KO+ MK2 vs MK2KO+vector)		1,000000	0,069401	0,018783	0,727071	0,000334	0,000013	0,001233	**Statistically Significant values (p<0.01) shaded**

Experiment 2

		Control	2,5µM 5-ITu	5µM 5-ITu	10µM 5-ITu	2,5µM 5-ITu+TNF	5µM 5-ITu+TNF	10µM 5-ITu+TNF	
MK2 KO+ MK2	Biol. Replicate 1	99,44	91,69	94,81	96,41	84,48	91,94	90,16	
	Biol. Replicate 2	104,33	88,06	93,80	86,47	82,12	91,00	99,56	
	Biol. Replicate 3	96,24	94,17	93,33	84,48	84,88	88,73	89,64	
MK2 KO+ vector	Biol. Replicate 1	96,20	84,99	79,72	82,84	72,62	49,99	49,96	
	Biol. Replicate 2	101,36	88,30	78,64	81,84	70,92	55,40	52,67	
	Biol. Replicate 3	102,44	86,42	81,31	76,85	72,64	53,76	48,28	
p-values (MK2KO+ MK2 vs MK2KO+vector)		1,000000	0,098197	0,000412	0,130062	0,000720	0,000157	0,002180	**Statistically Significant values (p<0.01) shaded**

Experiment 3

		Control	2,5µM 5-ITu	5µM 5-ITu	10µM 5-ITu	2,5µM 5-ITu+TNF	5µM 5-ITu+TNF	10µM 5-ITu+TNF	
MK2 KO+ MK2	Biol. Replicate 1	99,08	86,80	88,95	87,46	88,56	81,40	82,14	
	Biol. Replicate 2	101,06	88,55	88,59	89,94	90,19	80,62	80,16	
	Biol. Replicate 3	99,86	88,48	89,10	87,59	88,19	83,16	83,09	
MK2 KO+ vector	Biol. Replicate 1	92,65	73,70	74,54	72,67	71,57	33,35	32,36	
	Biol. Replicate 2	90,89	73,65	73,32	72,72	65,77	33,63	30,19	
	Biol. Replicate 3	89,44	74,28	73,07	65,99	64,26	32,72	28,30	
p-values (MK2KO+ MK2 vs MK2KO+vector)		0,002495	0,000553	0,000299	0,008789	0,006894	0,000049	0,000009	**Statistically Significant values (p<0.01) shaded**

Figure 3B

Representative experiment

		Control-6h	5-ITu-6h	Control-24h	5-ITu-24h	
MK2 KO+ MK2	Biol. Replicate 1	95,67	87,18	95,88	37,04	
	Biol. Replicate 2	100,98	81,32	98,66	39,33	
	Biol. Replicate 3	103,35	78,88	105,46	38,92	
MK2 KO+ vector	Biol. Replicate 1	98,03	84,11	99,36	33,73	
	Biol. Replicate 2	101,84	82,65	101,01	33,16	
	Biol. Replicate 3	100,13	76,90	99,63	32,28	
p-values (MK2KO+ MK2 vs MK2KO+vector)		1,000000	0,727071	1,000000	0,005602	**Statistically Significant values (p<0.01) shaded**

Experiment 2

		Control-6h	5-ITu-6h	Control-24h	5-ITu-24h	
MK2 KO+ MK2	Biol. Replicate 1	95,67	94,07	100,24	26,41	
	Biol. Replicate 2	100,98	91,61	99,54	26,83	
	Biol. Replicate 3	103,35	90,52	100,22	27,03	
MK2 KO+ vector	Biol. Replicate 1	98,03	78,14	100,38	27,08	
	Biol. Replicate 2	101,84	79,32	100,83	26,11	
	Biol. Replicate 3	100,13	80,60	98,78	26,64	
p-values (MK2KO+ MK2 vs MK2KO+vector)		1,000000	0,001038	1,000000	0,695566	**Statistically Significant values (p<0.01) shaded**

Experiment 3

		Control-6h	5-ITu-6h	Control-24h	5-ITu-24h	
MK2 KO+ MK2	Biol. Replicate 1	99,08	87,46	96,09	33,69	
	Biol. Replicate 2	101,06	89,94	100,88	34,61	
	Biol. Replicate 3	99,86	87,59	103,03	34,19	
MK2 KO+ vector	Biol. Replicate 1	92,65	72,67	86,44	26,08	
	Biol. Replicate 2	90,89	72,72	84,08	25,32	
	Biol. Replicate 3	89,44	65,99	84,45	20,76	
p-values (MK2KO+ MK2 vs MK2KO+vector)		0,002495	0,008789	0,010939	0,023616	**Statistically Significant values (p<0.01) shaded**

Figure 3C

Representative experiment

		-TNF	+TNF	5-ITu	5-ITu+TNF	ABT702	ABT702+TNF	Gemcitabine	Gemcitabine+TNF	Etoposide	Etoposide+TNF	Doxorubicin	Doxorubicin+TNF	Staurosporine	Staurosporine+TNF
MK2 KO+ MK2	Biol. Replicate 1	99,50	92,75	91,17	80,92	96,80	90,14	97,80	77,33	94,05	76,83	79,56	66,06	45,46	20,79
	Biol. Replicate 2	107,65	89,41	92,62	81,14	92,38	85,12	103,49	102,29	83,23	80,17	70,31	66,12	45,17	21,50
	Biol. Replicate 3	92,85	97,70	90,21	87,34	98,47	86,75	103,27	90,30	88,31	78,01	74,00	65,76	40,21	20,01
MK2 KO+ vector	Biol. Replicate 1	88,84	88,93	71,37	56,00	95,29	80,87	98,33	78,31	92,55	67,37	72,80	65,69	45,19	19,98
	Biol. Replicate 2	101,64	91,58	73,63	55,47	92,51	81,46	95,33	75,90	90,90	67,71	77,31	64,11	40,34	18,22
	Biol. Replicate 3	90,15	81,59	85,78	53,25	94,80	87,39	85,13	80,12	86,54	62,61	72,54	55,60	40,56	18,31
p-values (MK2KO+ MK2 vs MK2KO+vector)		0,335681	0,200678	0,081022	0,002099	0,467410	0,191509	0,151841	0,239448	0,710128	0,005753	0,902943	0,313862	0,533718	0,058568

Experiment 2

		-TNF	+TNF	5-ITu	5-ITu+TNF	ABT702	ABT702+TNF	Gemcitabine	Gemcitabine+TNF	Etoposide	Etoposide+TNF	Doxorubicin	Doxorubicin+TNF	Staurosporine	Staurosporine+TNF
MK2 KO+ MK2	Biol. Replicate 1	97,79	95,09	94,63	80,55	84,23	84,44	106,91	90,78	85,10	93,94	88,81	83,78	50,67	25,20
	Biol. Replicate 2	100,13	93,34	98,32	80,89	85,19	86,25	104,49	93,66	99,65	89,26	86,32	81,65	55,39	24,93
	Biol. Replicate 3	102,08	94,99	91,05	79,06	96,22	84,47	101,61	91,02	91,10	88,88	89,03	87,02	52,13	24,93
MK2 KO+ vector	Biol. Replicate 1	97,42	84,49	91,79	42,10	88,20	82,18	95,94	86,50	83,05	85,51	79,07	78,08	47,92	22,60
	Biol. Replicate 2	102,40	88,88	90,94	44,24	90,00	82,49	96,83	84,18	91,45	88,65	86,52	77,90	49,50	21,89
	Biol. Replicate 3	100,18	90,21	87,96	42,42	89,36	86,39	96,98	82,10	87,27	79,44	84,50	81,86	49,64	21,22
p-values (MK2KO+ MK2 vs MK2KO+vector)		1,000000	0,050503	0,157617	0,000002	0,883674	0,429904	0,032325	0,010766	0,402570	0,138098	0,158803	0,076159	0,102864	0,012610

Experiment 3

		-TNF	+TNF	5-ITu	5-ITu+TNF	ABT702	ABT702+TNF	Gemcitabine	Gemcitabine+TNF	Etoposide	Etoposide+TNF	Doxorubicin	Doxorubicin+TNF	Staurosporine	Staurosporine+TNF
MK2 KO+ MK2	Biol. Replicate 1	100,12	101,98	88,83	91,92	97,00	102,86	98,78	106,61	70,58	71,01	88,65	88,36	40,63	20,67
	Biol. Replicate 2	98,36	102,38	92,47	92,13	96,67	94,85	99,31	98,38	68,96	73,56	86,95	85,12	38,66	21,24
	Biol. Replicate 3	101,53	102,64	92,11	90,16	94,85	94,34	97,96	97,99	71,64	71,34	88,65	84,76	40,30	20,63
MK2 KO+ vector	Biol. Replicate 1	100,24	96,44	81,34	57,16	90,26	94,76	92,47	92,21	60,18	60,39	79,69	86,27	40,45	22,35
	Biol. Replicate 2	99,90	96,20	82,39	56,09	91,59	93,16	94,36	94,43	62,77	62,44	80,33	83,21	39,67	21,76
	Biol. Replicate 3	99,85	97,13	81,93	55,81	91,78	90,00	90,54	92,50	63,58	56,73	79,95	81,36	39,46	21,69
p-values (MK2KO+ MK2 vs MK2KO+vector)		1,000000	0,000163	0,011139	0,000006	0,005123	0,226766	0,020225	0,097913	0,003904	0,008306	0,002422	0,253213	0,997864	0,019770

Experiment 4

		-TNF	+TNF	5-ITu	5-ITu+TNF	ABT702	ABT702+TNF	Gemcitabine	Gemcitabine+TNF	Etoposide	Etoposide+TNF	Doxorubicin	Doxorubicin+TNF	Staurosporine	Staurosporine+TNF
MK2 KO+ MK2	Biol. Replicate 1	100,52	97,75	83,30	82,26	92,01	90,49	90,01	94,71	80,97	86,28	76,68	76,20	44,93	23,06
	Biol. Replicate 2	99,34	94,54	81,45	81,59	94,36	91,67	94,28	92,41	86,19	85,91	82,65	79,33	47,76	23,13

	Biol. Replicate 3	100,15	99,11	82,36	80,79	95,27	92,60	96,87	90,95	85,52	84,69	76,31	78,38	47,24	22,24
MK2 KO+ vector	Biol. Replicate 1	99,79	101,55	75,28	60,17	93,31	97,87	95,64	91,20	82,73	82,47	86,10	84,43	44,08	24,28
	Biol. Replicate 2	100,50	103,80	74,75	62,63	93,79	93,85	86,66	82,71	82,08	83,70	85,15	85,63	45,51	24,27
	Biol. Replicate 3	99,71	105,82	76,83	58,92	89,79	94,20	93,43	87,99	84,58	82,45	85,89	87,30	45,04	23,45
p-values (MK2KO+ MK2 vs MK2KO+vector)		1,000000	0,023137	0,001306	0,000863	0,379591	0,083333	0,621222	0,148533	0,589150	0,012807	0,070723	0,003425	0,169141	0,040101

Figure 3D

Representative experiment

		Control	5-ITu+TNF	5-ITu+TNF+Nec1s	
MK2 KO+ MK2	Biol. Replicate 1	99,44	90,16	90,22	
	Biol. Replicate 2	104,33	99,56	92,62	
	Biol. Replicate 3	96,24	89,64	93,28	
MK2 KO+ vector	Biol. Replicate 1	96,20	49,96	75,10	
	Biol. Replicate 2	101,36	52,67	75,35	
	Biol. Replicate 3	102,44	48,28	72,12	
p-value (5-ITu+TNF-MK2KO+ MK2 vs 5-ITu+TNF-MK2KO+vector)			0,002180		**Statistically Significant values (p<0.01) shaded**
p-value (5-ITu+TNF-MK2KO+vector vs 5-ITu+TNF+Nec1s-MK2KO+vector)				0,000170	

Experiment 2

		Control	5-ITu+TNF	5-ITu+TNF+Nec1s	
MK2 KO+ MK2	Biol. Replicate 1	97,79	80,55	87,94	
	Biol. Replicate 2	100,13	80,89	89,91	
	Biol. Replicate 3	102,08	79,06	94,41	
MK2 KO+ vector	Biol. Replicate 1	97,42	42,10	87,17	
	Biol. Replicate 2	102,40	44,24	91,08	
	Biol. Replicate 3	100,18	42,42	93,95	
p-value (5-ITu+TNF-MK2KO+ MK2 vs 5-ITu+TNF-MK2KO+vector)			0,000002		**Statistically Significant values (p<0.01) shaded**
p-value (5-ITu+TNF-MK2KO+vector vs 5-ITu+TNF+Nec1s-MK2KO+vector)				0,000622	

Experiment 3

		Control	5-ITu+TNF	5-ITu+TNF+Nec1s	
MK2 KO+ MK2	Biol. Replicate 1	96,27	83,02	92,21	
	Biol. Replicate 2	101,26	87,53	91,56	
	Biol. Replicate 3	102,47	89,55	90,03	
MK2 KO+ vector	Biol. Replicate 1	98,09	35,03	72,99	
	Biol. Replicate 2	99,82	36,52	83,33	
	Biol. Replicate 3	102,09	35,10	68,86	
p-value (5-ITu+TNF-MK2KO+ MK2 vs 5-ITu+TNF-MK2KO+vector)			0,000789		**Statistically Significant values (p<0.01) shaded**
p-value (5-ITu+TNF-MK2KO+vector vs 5-ITu+TNF+Nec1s-MK2KO+vector)				0,010906	

Figure 3E

Representative experiment

		Control	PF3644022	LPS+zVAD	LPS+zVAD+PF3644022	LPS+5-ITu	LPS+5-ITu+PF3644022	
RAW 264,7 macrophages	Biol. Replicate 1	105,14	77,54	100,58	12,70	51,66	14,94	
	Biol. Replicate 2	97,96	76,21	93,80	11,74	57,22	13,40	
	Biol. Replicate 3	96,90	69,93	95,29	11,23	55,72	14,07	
p-value (Control vs LPS+zVAD+PF3644022)					0,000647			**Statistically Significant values (p<0.01) shaded**
p-value (Control vs LPS+5-Itu)						0,000337		
p-value (Control vs LPS+5-Itu+PF3644022)							0,000669	

Experiment 2

		Control	PF3644022	LPS+zVAD	LPS+zVAD+PF3644022	LPS+5-ITu	LPS+5-ITu+PF3644022	
RAW 264,7 macrophages	Biol. Replicate 1	103,90	67,85	114,37	22,81	61,19	14,99	
	Biol. Replicate 2	98,25	66,64	106,83	21,51	60,15	15,74	
	Biol. Replicate 3	97,86	64,87	113,02	24,23	59,36	15,71	
p-value (Control vs LPS+zVAD+PF3644022)					0,000122			**Statistically Significant values (p<0.01) shaded**
p-value (Control vs LPS+5-Itu)						0,001312		
p-value (Control vs LPS+5-Itu+PF3644022)							0,000446	

Experiment 3

		Control	PF3644022	LPS+zVAD	LPS+zVAD+PF3644022	LPS+5-ITu	LPS+5-ITu+PF3644022	
RAW 264,7 macrophages	Biol. Replicate 1	100,08	70,08	107,77	16,82	60,38	14,65	
	Biol. Replicate 2	96,64	67,27	113,33	16,83	58,69	14,56	
	Biol. Replicate 3	103,28	69,19	112,28	16,71	61,84	16,92	
p-value (Control vs LPS+zVAD+PF3644022)					0,000528			**Statistically Significant values (p<0.01) shaded**
p-value (Control vs LPS+5-Itu)						0,000448		
p-value (Control vs LPS+5-Itu+PF3644022)							0,000091	

Experiment 4

		Control	PF3644022	LPS+zVAD	LPS+zVAD+PF3644022	LPS+5-ITu	LPS+5-ITu+PF3644022	
RAW 264,7 macrophages	Biol. Replicate 1	101,64	64,23	124,71	15,48	63,19	17,59	
	Biol. Replicate 2	96,38	65,21	114,26	17,88	62,19	19,92	
	Biol. Replicate 3	101,98	65,57	111,15	17,68	66,98	19,71	
p-value (Control vs LPS+zVAD+PF3644022)					0,000070			**Statistically Significant values (p<0.01) shaded**
p-value (Control vs LPS+5-Itu)						0,000138		
p-value (Control vs LPS+5-Itu+PF3644022)							0,000082	

Figure 3F

Representative experiment

		Control	5-ITu	5-ITu+ PF3644022	5-ITu+ PF3644022+Nec1s	SM+5-ITu	SM+5-ITu+PF3644022	M+5-ITu+PF3644022+Nec1s	
RAW 264,7 macrophages	Biol. Replicate 1	98,34	80,21	74,90	72,62	68,80	50,47	75,43	
	Biol. Replicate 2	98,34	75,79	74,09	69,85	65,93	48,56	73,55	
	Biol. Replicate 3	103,31	72,25	69,73	70,06	66,39	48,76	74,37	
p-value (SM+5-ITu vs SM+5-ITu+PF3644022)							0,000183		**Statistically Significant values (p<0.01) shaded**
p-value (SM+5-ITu+PF3644022 vsSM+5-ITu+PF3644022+Nec1s)								0,000007	

Experiment 2

		Control	5-ITu	5-ITu+ PF3644022	5-ITu+ PF3644022+Nec1s	SM+5-ITu	SM+5-ITu+PF3644022	M+5-ITu+PF3644022+Nec1s	
RAW 264,7 macrophages	Biol. Replicate 1	103,90	55,20	22,03	28,01	47,16	9,16	29,15	
	Biol. Replicate 2	98,25	58,57	21,26	29,48	47,85	7,23	23,93	
	Biol. Replicate 3	97,86	56,80	24,57	30,19	44,42	7,58	22,27	
p-value (SM+5-ITu vs SM+5-ITu+PF3644022)							0,000044		**Statistically Significant values (p<0.01) shaded**
p-value (SM+5-ITu+PF3644022 vsSM+5-ITu+PF3644022+Nec1s)								0,0098204	

Experiment 3

		Control	5-ITu	5-ITu+ PF3644022	5-ITu+ PF3644022+Nec1s	SM+5-ITu	SM+5-ITu+PF3644022	M+5-ITu+PF3644022+Nec1s	
RAW 264,7 macrophages	Biol. Replicate 1	100,08	65,94	25,26	33,44	45,33	6,56	31,05	
	Biol. Replicate 2	96,64	62,70	24,41	36,30	44,52	6,61	29,91	
	Biol. Replicate 3	103,28	60,97	29,70	35,25	46,86	5,48	29,19	
p-value (SM+5-ITu vs SM+5-ITu+PF3644022)							0,000014		**Statistically Significant values (p<0.01) shaded**

p-value (SM+5-ITu+PF3644022 vsSM+5-ITu+PF3644022+Nec1s)									0,0000112	
**Experiment 4**			Control	5-ITu	5-ITu+ PF3644022	5-ITu+ PF3644022+Nec1s	SM+5-ITu	SM+5-ITu+PF3644022	M+5-ITu+PF3644022+Nec1s	
	RAW 264,7 macrophages	Biol. Replicate 1	101,64	66,13	27,82	40,97	54,14	9,83	34,04	
		Biol. Replicate 2	96,38	62,10	27,18	48,37	52,79	8,15	34,34	
		Biol. Replicate 3	101,98	65,40	33,35	50,21	53,93	8,43	36,09	
p-value (SM+5-ITu vs SM+5-ITu+PF3644022)								0,000000		**Statistically Significant values (p<0.01) shaded**
p-value (SM+5-ITu+PF3644022 vsSM+5-ITu+PF3644022+Nec1s)									0,0000090	

Supplementary Figure 2a

			Control	2,5µM 5-ITu	5µM 5-ITu	10µM 5-ITu	2,5µM 5-ITu+TNF	5µM 5-ITu+TNF	10µM 5-ITu+TNF	
**Representative experiment**			Control	2,5µM 5-ITu	5µM 5-ITu	10µM 5-ITu	2,5µM 5-ITu+TNF	5µM 5-ITu+TNF	10µM 5-ITu+TNF	
	DKO+ MK2	Biol. Replicate 1	91,80	101,02	93,42	103,22	108,61	81,87	93,73	
		Biol. Replicate 2	105,41	104,40	102,93	102,65	109,30	94,44	91,81	
		Biol. Replicate 3	102,79	102,13	104,38	102,34	117,12	96,84	92,31	
	DKO+ vector	Biol. Replicate 1	106,27	104,20	112,23	103,69	83,35	46,61	46,72	
		Biol. Replicate 2	105,50	105,50	105,36	101,72	82,02	46,24	42,46	
		Biol. Replicate 3	107,10	102,24	115,95	96,34	77,28	50,04	40,81	
		p-values (DKO+ MK2 vs DKO+vector)	0,269567	0,346878	0,078200	0,430599	0,001327	0,007939	0,000475	**Statistically Significant values (p<0.01) shaded**
**Experiment 2**			Control	2,5µM 5-ITu	5µM 5-ITu	10µM 5-ITu	2,5µM 5-ITu+TNF	5µM 5-ITu+TNF	10µM 5-ITu+TNF	
	DKO+ MK2	Biol. Replicate 1	95,74	101,57	96,20	94,02	100,28	92,60	85,81	
		Biol. Replicate 2	99,82	97,48	96,40	95,21	98,72	96,26	86,82	
		Biol. Replicate 3	104,44	94,80	95,64	93,91	96,69	90,06	85,38	
	DKO+ vector	Biol. Replicate 1	99,60	94,43	97,58	94,09	94,05	76,33	62,97	
		Biol. Replicate 2	101,49	96,48	96,96	89,10	83,76	75,66	61,16	
		Biol. Replicate 3	98,91	99,01	96,48	91,99	82,24	72,73	56,12	
		p-values (DKO+ MK2 vs DKO+vector)	1,000000	0,614365	0,084126	0,202432	0,075665	0,002257	0,004688	**Statistically Significant values (p<0.01) shaded**
**Experiment 3**			Control	2,5µM 5-ITu	5µM 5-ITu	10µM 5-ITu	2,5µM 5-ITu+TNF	5µM 5-ITu+TNF	10µM 5-ITu+TNF	
	DKO+ MK2	Biol. Replicate 1	102,17	91,95	97,16	87,27	96,08	88,39	80,06	
		Biol. Replicate 2	98,12	94,12	99,64	85,89	91,39	86,19	75,40	
		Biol. Replicate 3	99,71	92,77	101,07	86,46	91,18	88,06	75,38	
	DKO+ vector	Biol. Replicate 1	96,00	91,35	100,27	84,31	79,04	60,13	51,25	
		Biol. Replicate 2	104,09	94,25	94,07	85,34	78,55	64,11	52,00	
		Biol. Replicate 3	99,92	88,77	92,78	82,11	77,74	62,31	52,82	
		p-values (DKO+ MK2 vs DKO+vector)	1,000000	0,454594	0,260824	0,094940	0,009095	0,000192	0,002114	**Statistically Significant values (p<0.01) shaded**

Supplementary Figure 2b

Representative experiment

**Experiment 1**			Control-6h	5-ITu-6h	Control-24h	5-ITu-24h	
	DKO+ MK2	Biol. Replicate 1	91,80	103,22	94,92	21,85	
		Biol. Replicate 2	105,41	102,65	103,20	20,88	
		Biol. Replicate 3	102,79	102,34	101,87	20,68	
	DKO+ vector	Biol. Replicate 1	106,27	103,69	102,66	20,43	
		Biol. Replicate 2	105,50	101,72	98,83	21,21	
		Biol. Replicate 3	107,10	96,34	98,51	19,81	
		p-values (DKO+ MK2 vs DKO+vector)	0,269567	0,430599	1,000000	0,296533	**Statistically Significant values (p<0.01) shaded**
**Experiment 2**			Control-6h	5-ITu-6h	Control-24h	5-ITu-24h	
	DKO+ MK2	Biol. Replicate 1	102,17	87,27	97,66	19,72	
		Biol. Replicate 2	98,12	85,89	100,30	19,18	
		Biol. Replicate 3	99,71	86,46	102,04	18,49	
	DKO+ vector	Biol. Replicate 1	96,00	84,31	98,77	27,27	
		Biol. Replicate 2	104,09	85,34	102,62	26,68	
		Biol. Replicate 3	99,92	82,11	98,61	26,79	
		p-values (DKO+ MK2 vs DKO+vector)	1,000000	0,094940	1,000000	0,000310	**Statistically Significant values (p<0.01) shaded**

Supplementary Figure 2c

Representative experiment

**Experiment 1**			Control	TNF	5-ITu	5-ITu+TNF	ABT702	ABT702+TNF	Gemcitabine	Gemcitabine+TNF	Etoposide	Etoposide+TNF	Doxorubicin	Doxorubicin+TNF	Staurosporine	Staurosporine+TNF
	DKO+ MK2	Biol. Replicate 1	97,510	99,41	95,60	91,16	107,13	95,601	106,99	85,81	71,82	57,58	89,93	79,83	49,08	27,42
		Biol. Replicate 2	101,036	97,04	95,89	91,70	105,79	95,267	103,27	86,05	69,53	57,27	86,05	82,26	46,23	27,72
		Biol. Replicate 3	101,454	99,41	95,05	95,31	101,73	101,425	104,99	86,49	68,92	57,43	88,71	87,88	47,84	28,16
	DKO+ vector	Biol. Replicate 1	99,90	96,35	97,76	64,60	90,46	101,99	94,07	84,81	70,41	51,26	89,88	90,60	49,094	32,77
		Biol. Replicate 2	100,85	99,89	96,82	59,86	90,05	98,54	95,87	89,37	69,48	50,99	88,33	91,96	48,536	32,64
		Biol. Replicate 3	99,25	101,03	97,45	59,91	90,18	96,49	89,00	86,68	68,65	48,92	89,73	87,53	49,580	32,07
		p-values (DKO+ MK2 vs DKO+vector)	1,000000	0,789499	0,008126	0,000132	0,011695	0,572658	0,013497	0,594908	0,607559	0,010019	0,455449	0,087453	0,237406	0,000100
**Experiment 2**			Control	TNF	5-ITu	5-ITu+TNF	ABT702	ABT702+TNF	Gemcitabine	Gemcitabine+TNF	Etoposide	Etoposide+TNF	Doxorubicin	Doxorubicin+TNF	Staurosporine	Staurosporine+TNF
	DKO+ MK2	Biol. Replicate 1	95,88	93,82	91,80	92,44	92,82	100,34	90,38	81,17	77,37	72,45	81,83	81,39	43,48	26,24
		Biol. Replicate 2	100,63	93,18	92,68	87,31	104,74	97,34	99,27	94,62	75,91	70,20	81,33	78,69	44,17	25,92
		Biol. Replicate 3	103,50	94,17	95,79	88,89	105,63	95,78	100,07	85,35	76,90	71,81	83,28	80,71	44,87	25,74
	DKO+ vector	Biol. Replicate 1	101,58	99,51	95,61	63,33	96,57	100,41	85,81	88,64	72,16	58,76	81,88	80,06	48,79	29,01
		Biol. Replicate 2	100,26	101,79	89,01	62,76	96,40	99,18	88,25	89,53	75,47	58,63	88,08	82,03	48,13	29,59
		Biol. Replicate 3	98,16	99,77	96,01	57,11	99,63	99,88	92,90	88,30	75,91	59,55	88,20	81,43	49,60	29,47
		p-values (DKO+ MK2 vs DKO+vector)	1,000000	0,005403	0,965605	0,000484	0,485982	0,270876	0,122257	0,698447	0,193627	0,000757	0,196120	0,421461	0,001361	0,000156

Supplementary Figure 2d

Representative experiment

**Experiment 1**			Control	5-ITu+TNF	5-ITu+TNF+Nec1s	
	DKO+ MK2	Biol. Replicate 1	102,17	80,06	86,57	
		Biol. Replicate 2	98,12	75,40	83,05	
		Biol. Replicate 3	99,71	75,38	84,75	
	DKO+ vector	Biol. Replicate 1	96,00	51,25	76,47	
		Biol. Replicate 2	104,09	52,00	74,34	
		Biol. Replicate 3	99,92	52,82	71,27	
p-values (5-ITu +TNF-DKO+MK2 vs 5-ITu +TNF-DKO+vector)				0,0021143		**Statistically Significant values (p<0.01) shaded**
p-values (5-ITu +TNF-DKO+MK2 vs 5-ITu +TNF+Nec1s-DKO+vector)					0,002542	
**Experiment 2**			Control	5-ITu+TNF	5-ITu+TNF+Nec1s	
	DKO+ MK2	Biol. Replicate 1	94,05	80,88	95,99	
		Biol. Replicate 2	104,13	88,09	93,14	
		Biol. Replicate 3	101,82	87,39	95,34	

	DKO+ vector	Biol. Replicate 1	98,15	44,52	83,71				
		Biol. Replicate 2	103,12	42,44	77,81				
		Biol. Replicate 3	98,73	43,03	71,43				
p-values (5-ITu +TNF-DKO+MK2 vs 5-ITu +TNF-DKO+vector)				0,0016968		**Statistically Significant values (p<0.01) shaded**			
p-values (5-ITu +TNF-DKO+MK2 vs 5-ITu +TNF+Nec1s-DKO+vector)					0,008911				
