# Supplementary material for: 5-Iodotubercidin sensitizes cells to RIPK1-dependent necroptosis by interfering with NFκB signaling
Source: Cell Death Discov. 2023 Jul 26;9:262. doi: 10.1038/s41420-023-01576-x (PMC10372004; doi:10.1038/s41420-023-01576-x)
Supplement: Supplementary file 7 — Original Data File [file 41420_2023_1576_MOESM7_ESM.pptx]

## Slide 1
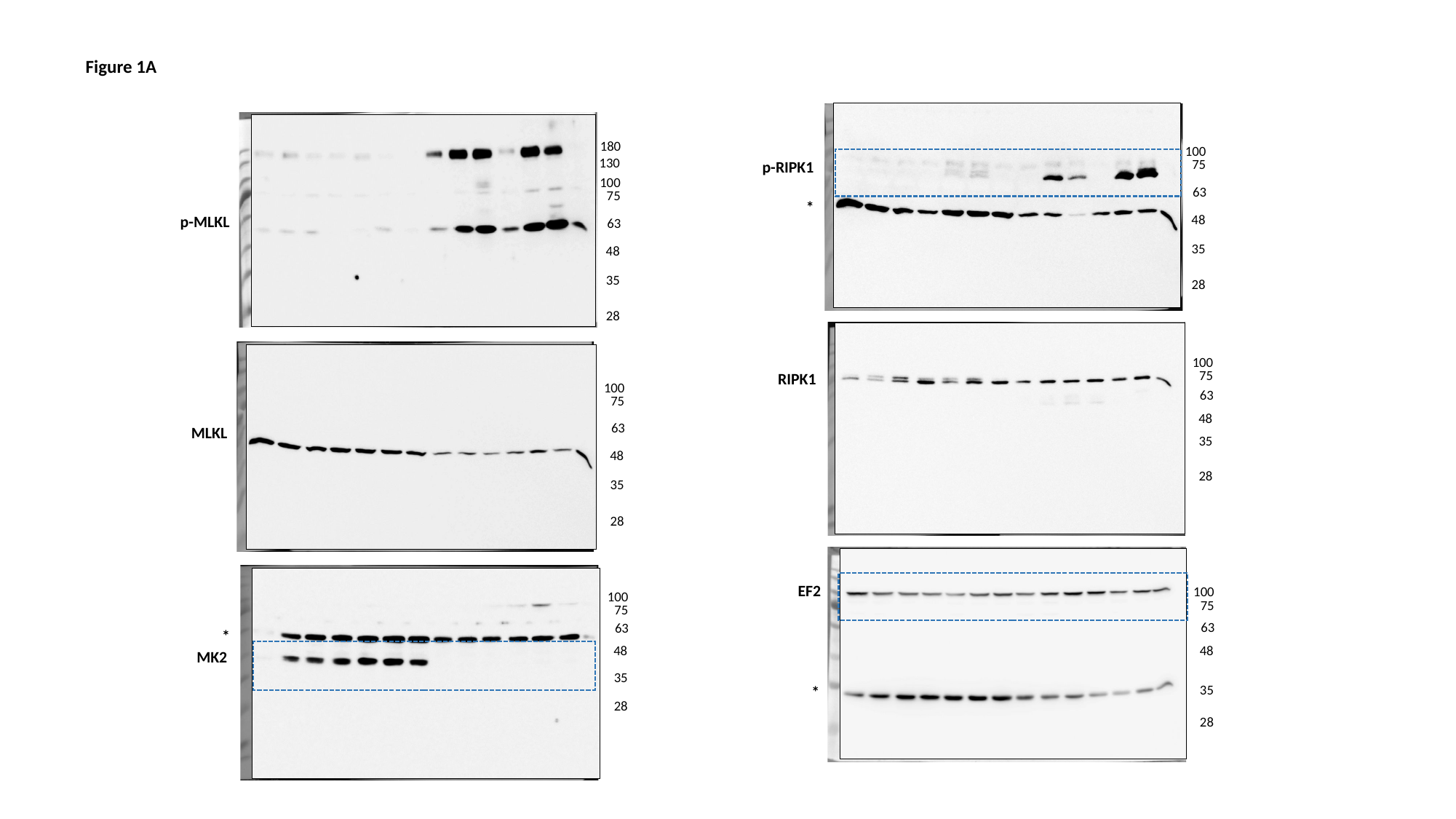

Figure 1A
180
100
75
63
48
35
28
130
p-RIPK1
100
75
63
48
35
28
*
p-MLKL
100
75
63
48
35
28
RIPK1
100
75
63
48
35
28
MLKL
EF2
100
75
63
48
35
28
*
100
75
63
48
35
28
*
MK2

## Slide 2
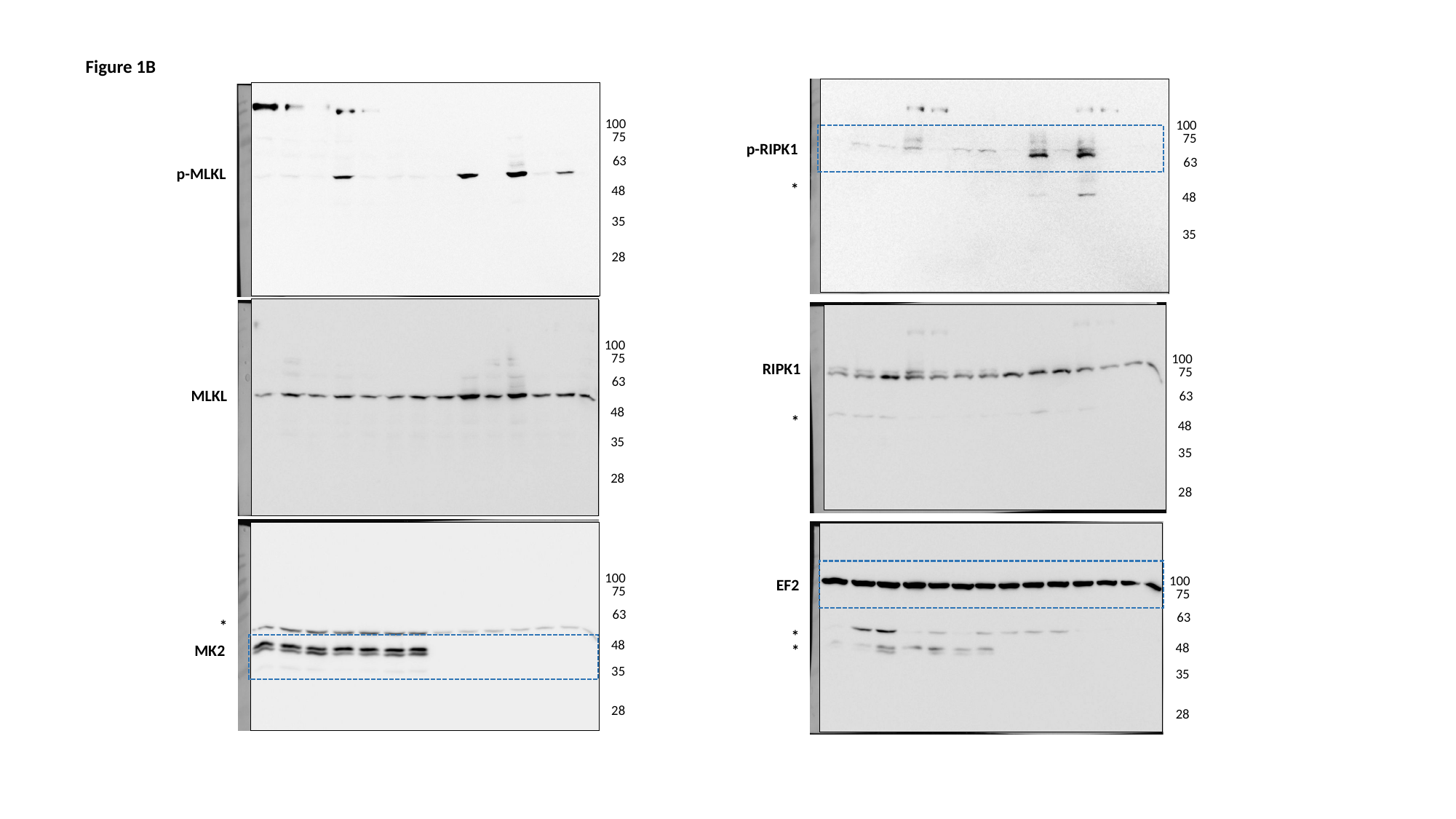

Figure 1B
100
75
63
48
35
28
100
75
63
48
35
p-RIPK1
p-MLKL
*
100
75
63
48
35
28
100
75
63
48
35
28
RIPK1
MLKL
*
*
MK2
100
75
63
48
35
28
100
75
63
48
35
28
EF2
*
*

## Slide 3
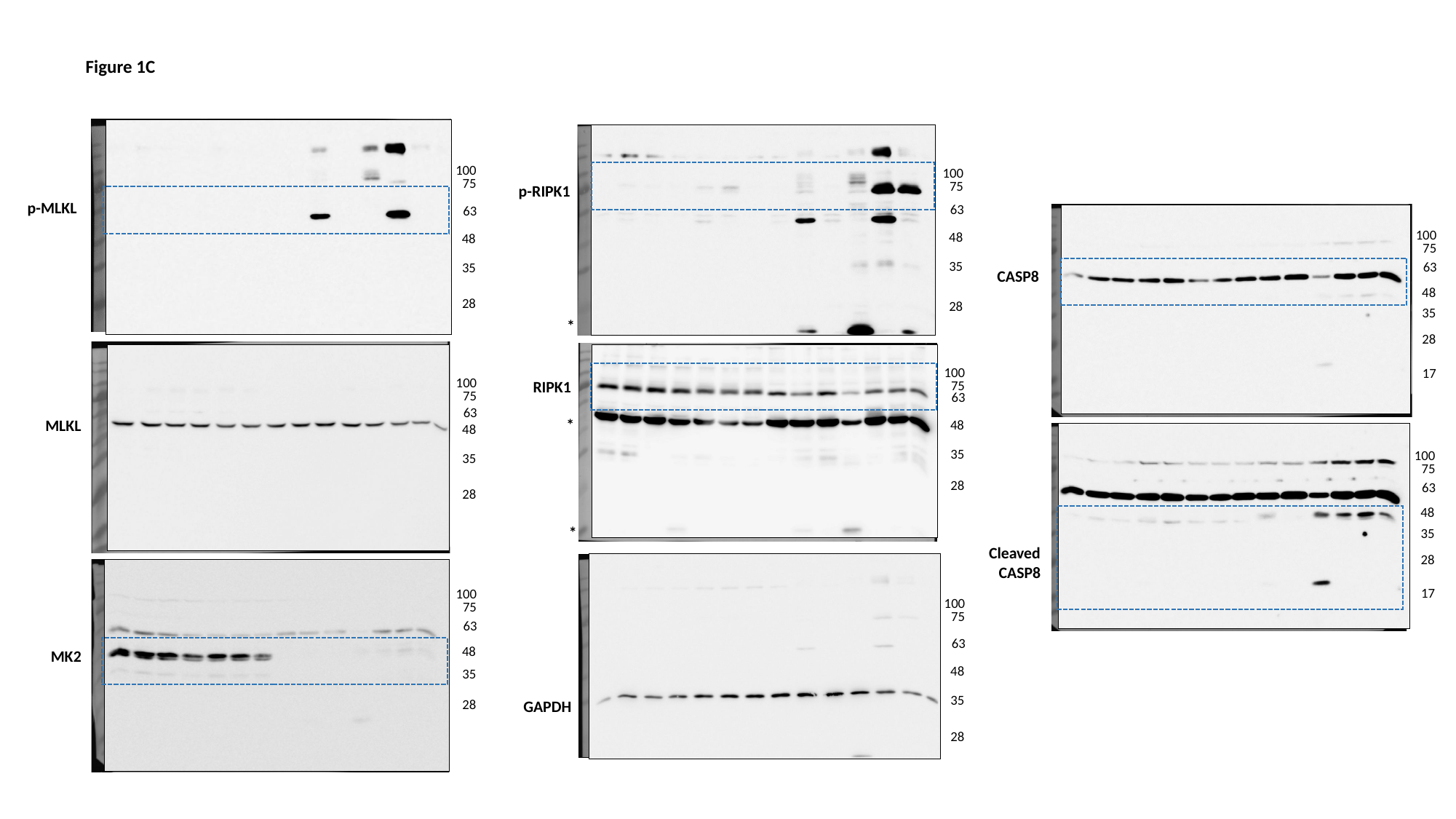

Figure 1C
100
75
63
48
35
28
100
75
63
48
35
28
p-RIPK1
p-MLKL
100
75
63
48
35
28
17
CASP8
*
100
75
63
48
35
28
100
75
63
48
35
28
RIPK1
*
MLKL
100
75
63
48
35
28
17
*
Cleaved CASP8
100
75
63
48
35
28
100
75
63
48
35
28
MK2
GAPDH

## Slide 4
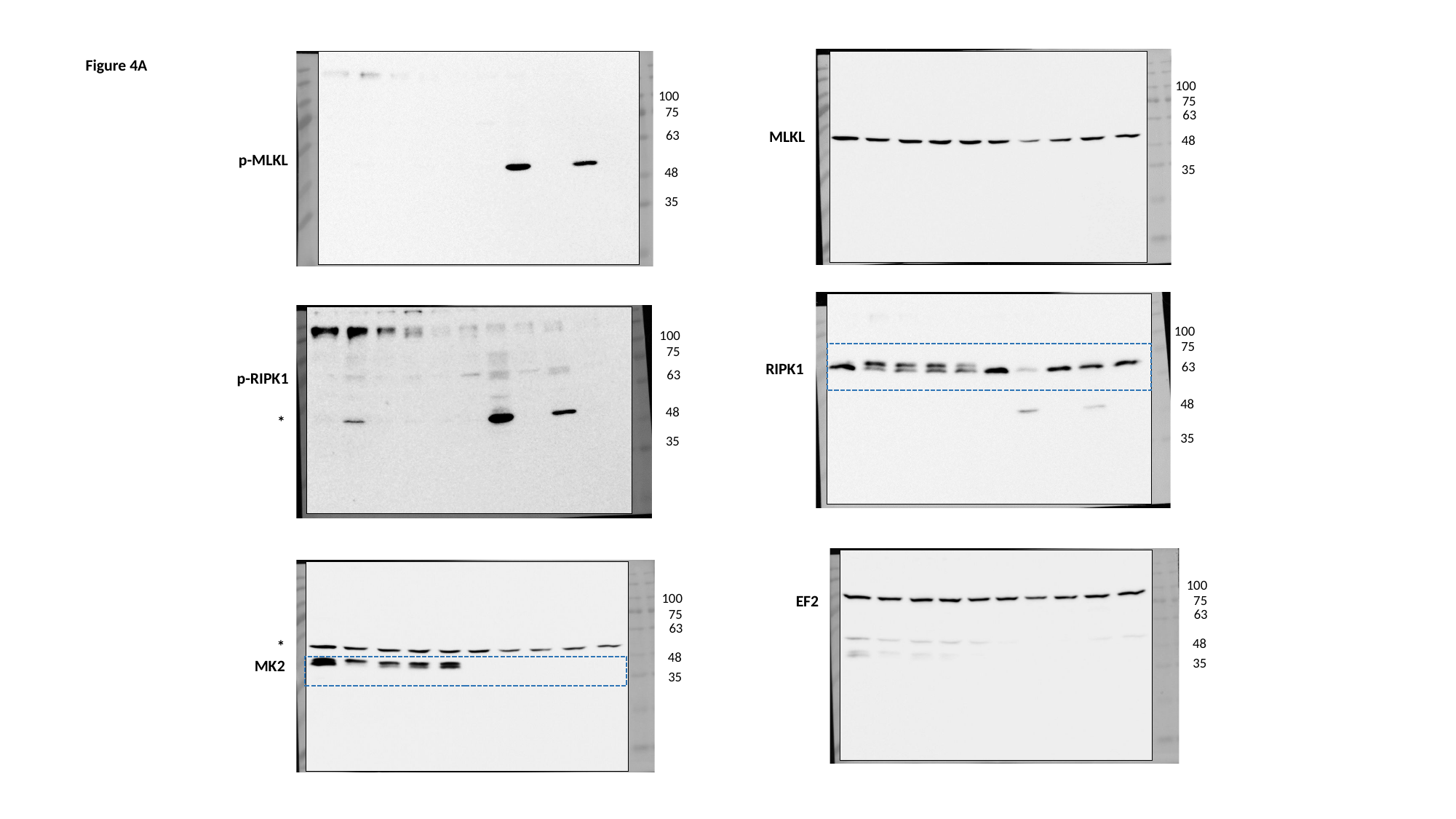

Figure 4A
100
75
63
48
35
100
75
63
48
35
MLKL
p-MLKL
100
75
63
48
35
100
75
63
48
35
RIPK1
p-RIPK1
*
100
75
63
48
35
100
75
63
48
35
EF2
*
MK2

## Slide 5
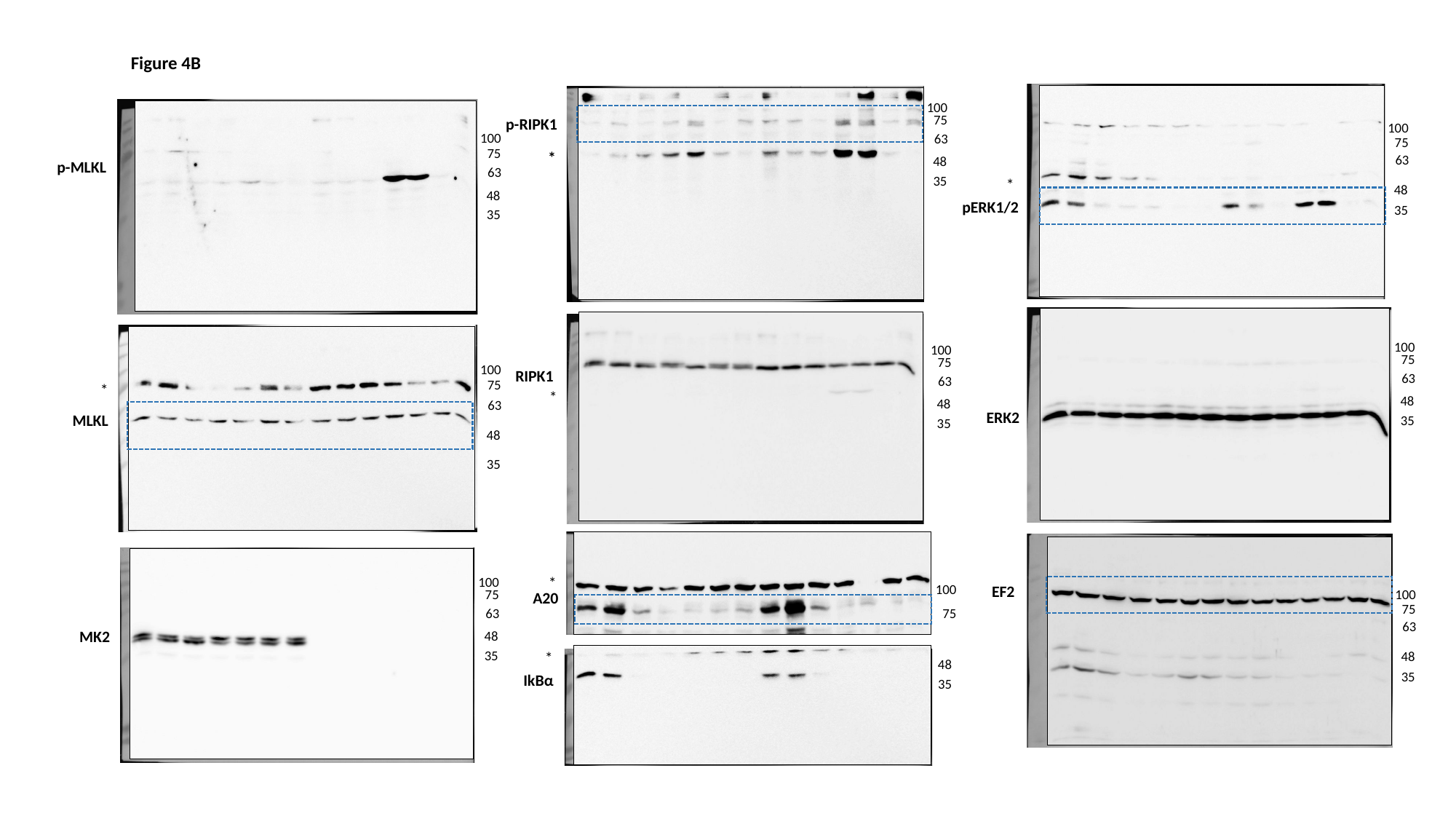

Figure 4B
100
75
63
48
35
p-MLKL
*
MLKL
MK2
p-RIPK1
100
75
63
48
35
100
75
63
48
35
*
*
pERK1/2
100
75
63
48
35
100
75
63
48
35
100
75
63
48
35
RIPK1
*
ERK2
*
100
75
63
48
35
100
75
 EF2
100
75
63
48
35
A20
*
 IkBα
48
35

## Slide 6
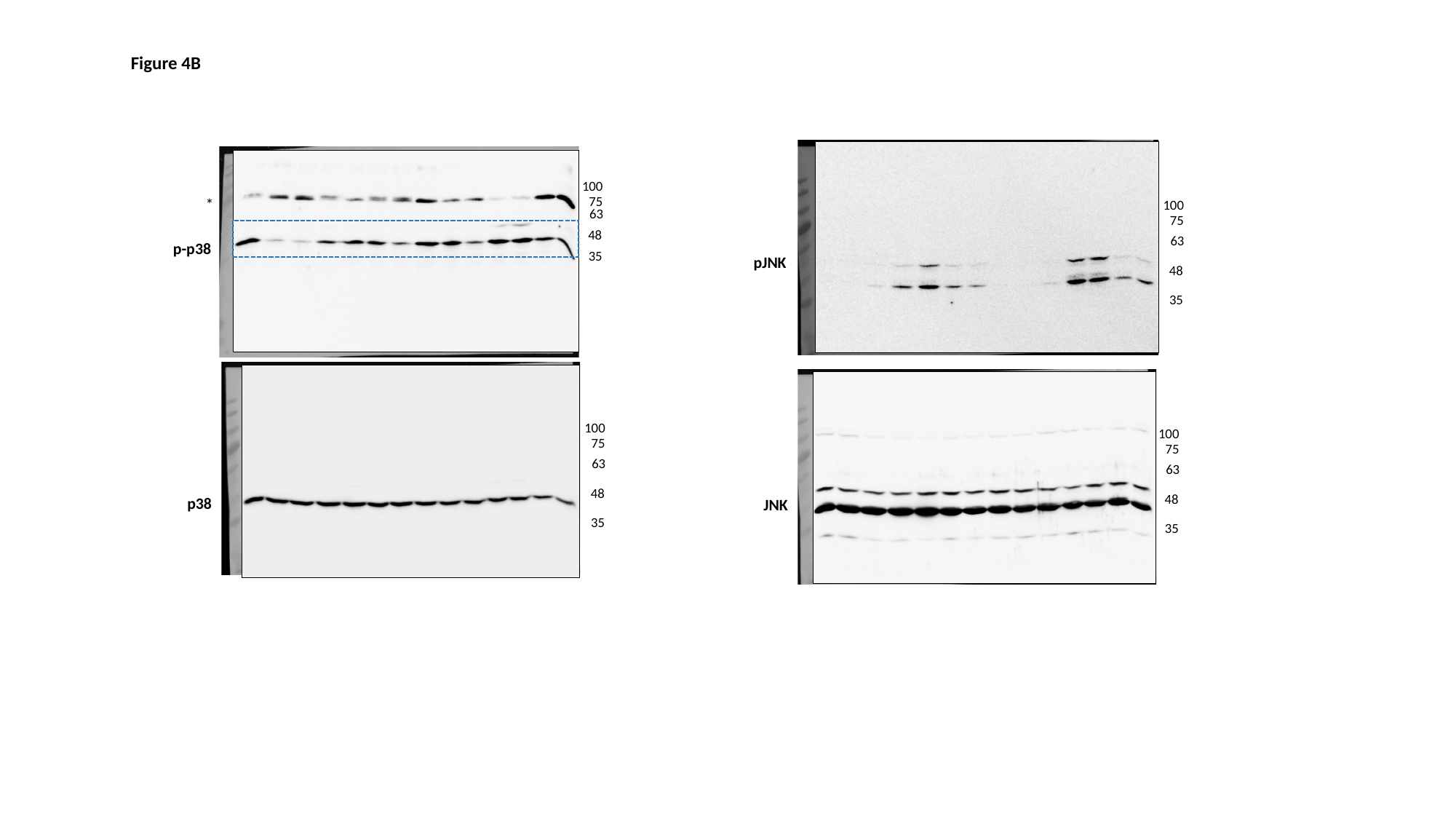

Figure 4B
100
75
63
48
35
*
100
75
63
48
35
p-p38
pJNK
100
75
63
48
35
100
75
63
48
35
p38
JNK

## Slide 7
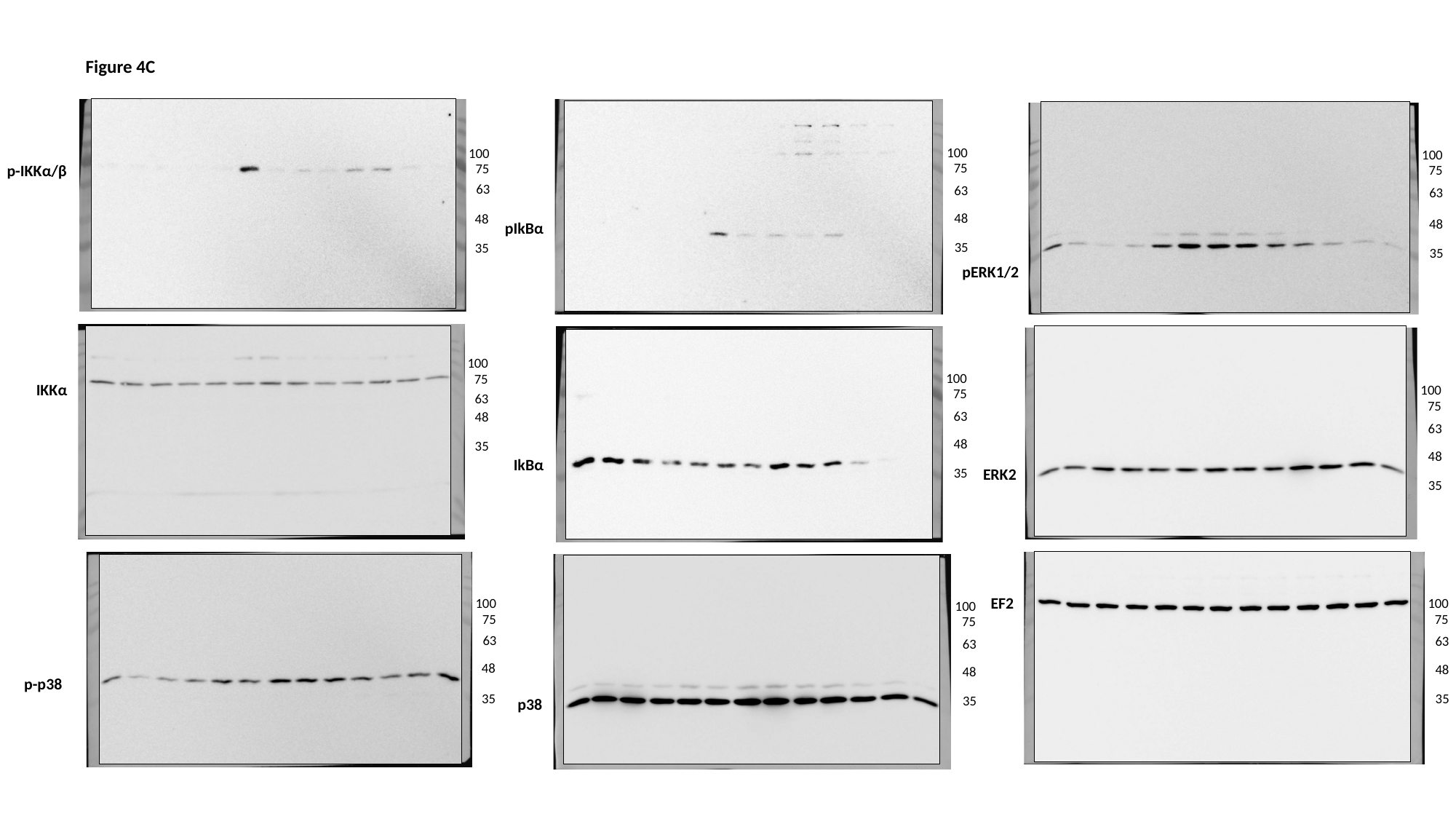

Figure 4C
 pIkBα
pERK1/2
100
75
63
48
35
100
75
63
48
35
100
75
63
48
35
p-IKKα/β
IKKα
ERK2
 IkBα
100
75
63
48
35
100
75
63
48
35
100
75
63
48
35
 EF2
p38
100
75
63
48
35
100
75
63
48
35
100
75
63
48
35
p-p38

## Slide 8
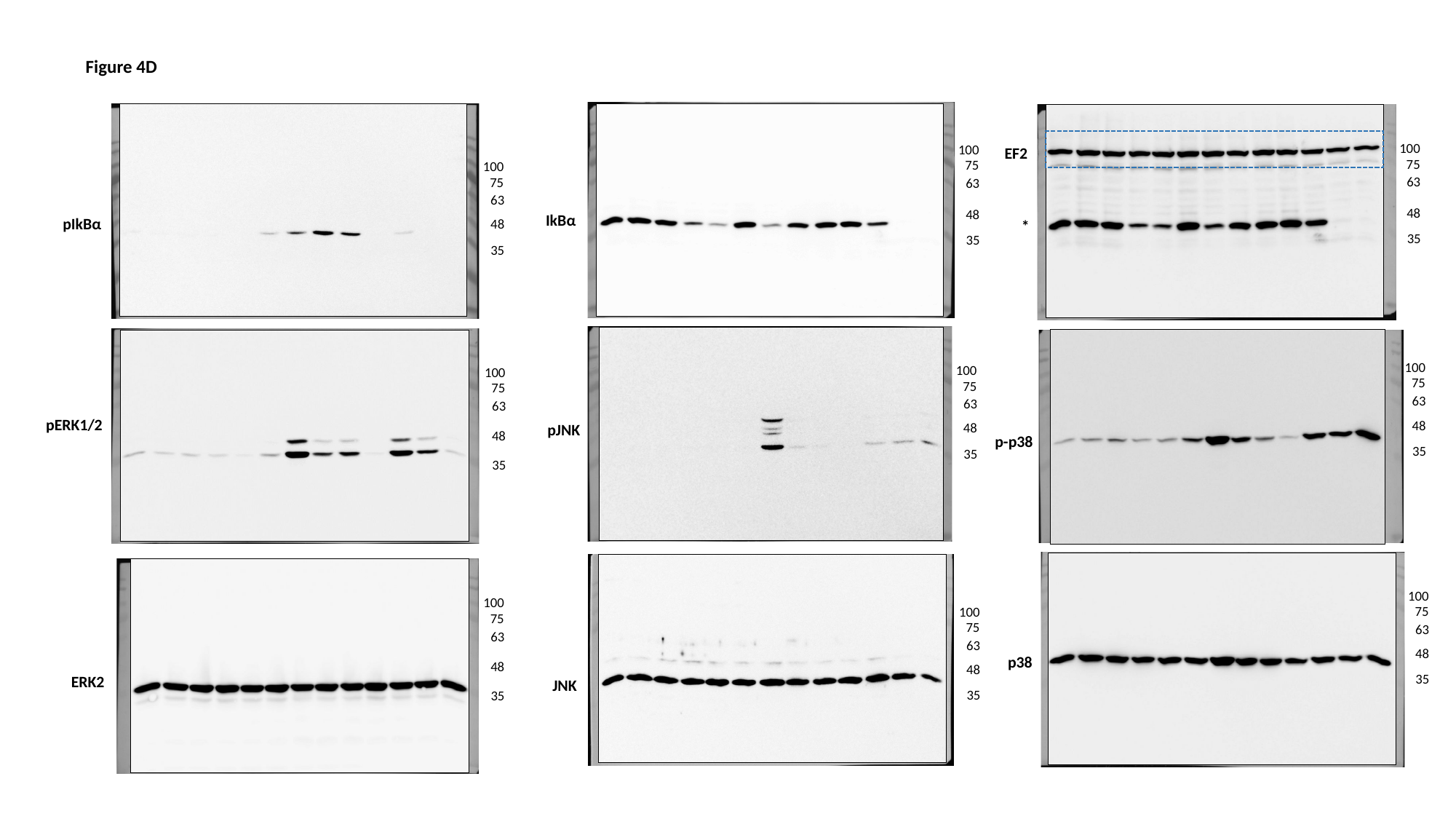

Figure 4D
100
75
63
48
35
100
75
63
48
35
 EF2
100
75
63
48
35
 IkBα
 pIkBα
 *
100
75
63
48
35
100
75
63
48
35
100
75
63
48
35
pERK1/2
pJNK
p-p38
100
75
63
48
35
100
75
63
48
35
100
75
63
48
35
p38
ERK2
JNK

## Slide 9
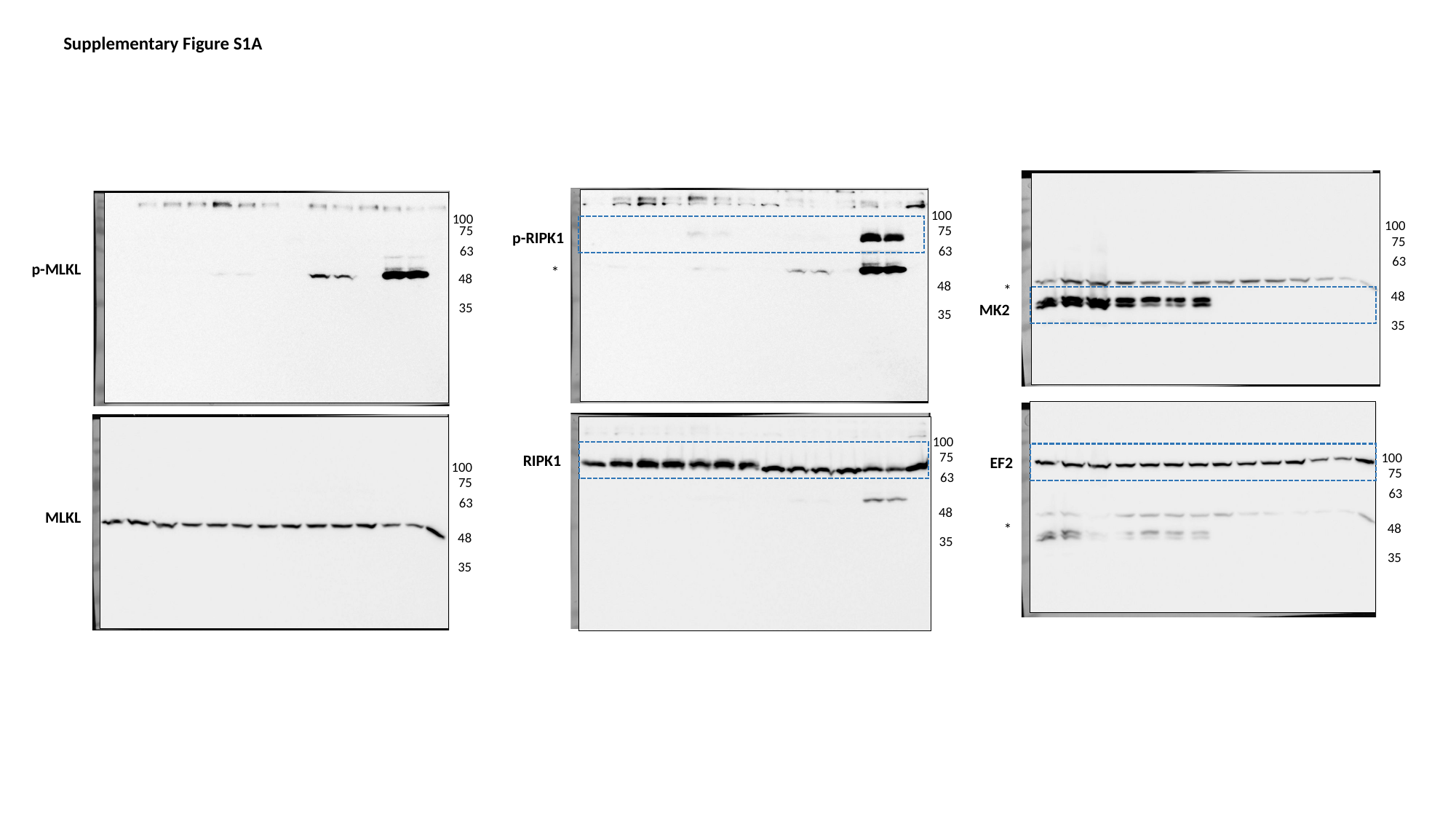

Supplementary Figure S1A
100
75
63
48
35
100
75
63
48
35
100
75
63
48
35
p-RIPK1
p-MLKL
*
*
MK2
100
75
63
48
35
100
75
63
48
35
RIPK1
 EF2
100
75
63
48
35
MLKL
*

## Slide 10
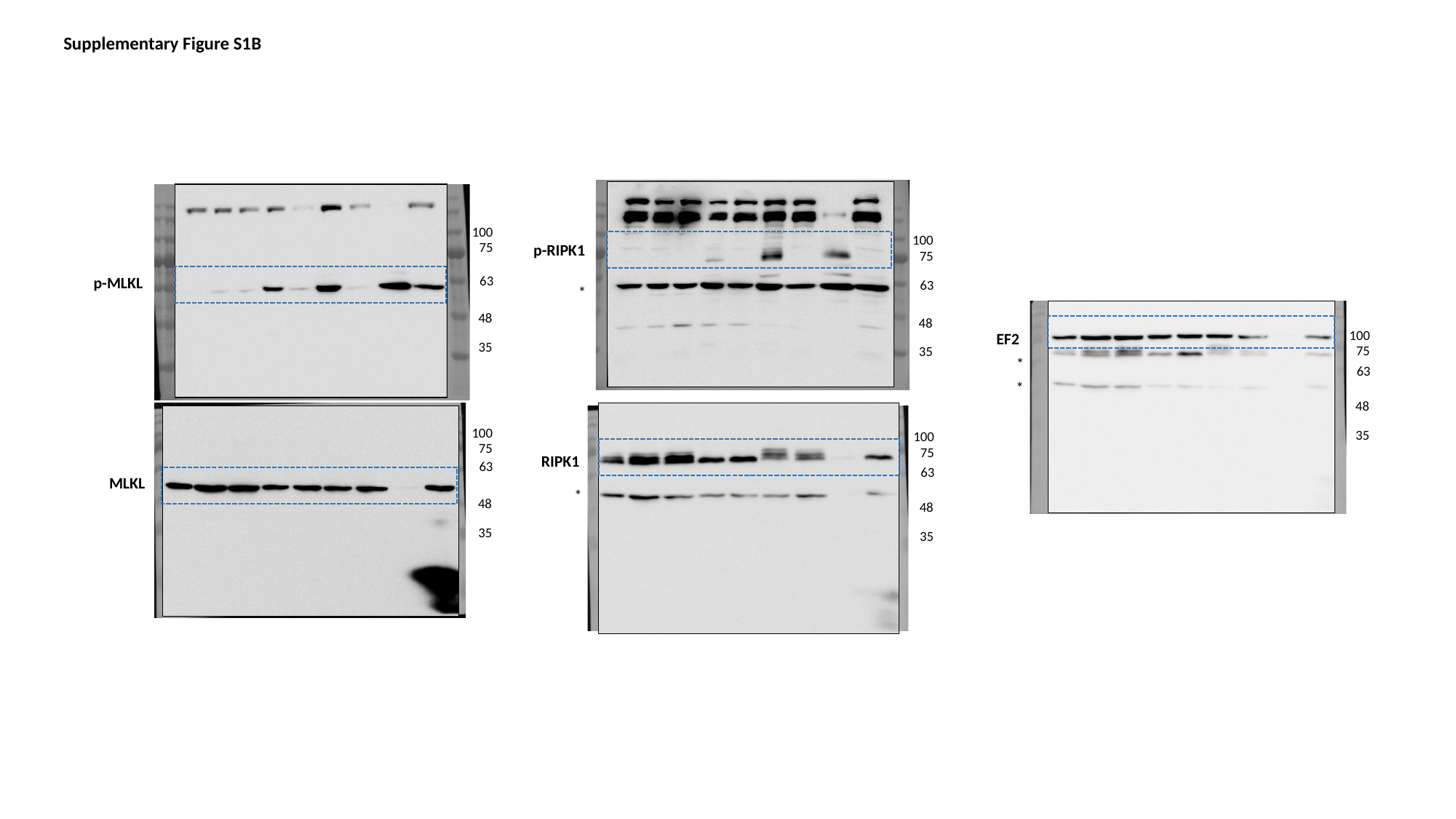

Supplementary Figure S1B
100
75
63
48
35
100
75
63
48
35
p-RIPK1
p-MLKL
*
100
75
63
48
35
EF2
*
*
100
75
63
48
35
100
75
63
48
35
RIPK1
MLKL
*
